# Supplementary material for: Cooperative catalysis by a single-atom enzyme-metal complex
Source: Nat Commun. 2022 Apr 21;13:2189. doi: 10.1038/s41467-022-29900-6 (PMC9023488; doi:10.1038/s41467-022-29900-6)
Supplement: Supplementary file 1 — Supplementary Information [file 41467_2022_29900_MOESM1_ESM.pdf]

Supplementary Information

Cooperative catalysis by a single-atom enzyme-metal complex

Xiaoyang Li[1, 2†], Yufei Cao[1†], Kai Luo[3], Lin Zhang[4*], Yunxiu Bai[1], Jiarong Xiong[1], Richard N. Zare[3*], Jun Ge[1, 5*]

1 Key Lab for Industrial Biocatalysis, Ministry of Education, Department of Chemical Engineering, Tsinghua University, Beijing 100084, China.

2 State Key Laboratory of Food Science and Technology, School of Food Science and Technology, Nanchang University, Nanchang 330047, China.

3 Department of Chemistry, Fudan University, Jiangwan Campus, Shanghai 200438, China.

4 Department of Biochemical Engineering and Key Laboratory of Systems Bioengineering of the Ministry of Education, School of Chemical Engineering and Technology, Tianjin University, Tianjin 300350, China.

5 Institute of Biopharmaceutical and Health Engineering, Tsinghua Shenzhen International Graduate School, Shenzhen 518055, China.

†These authors contributed equally to this work.

*Corresponding author. Email: junge@mail.tsinghua.edu.cn; zare@stanford.edu; linzhang@tju.edu.cn

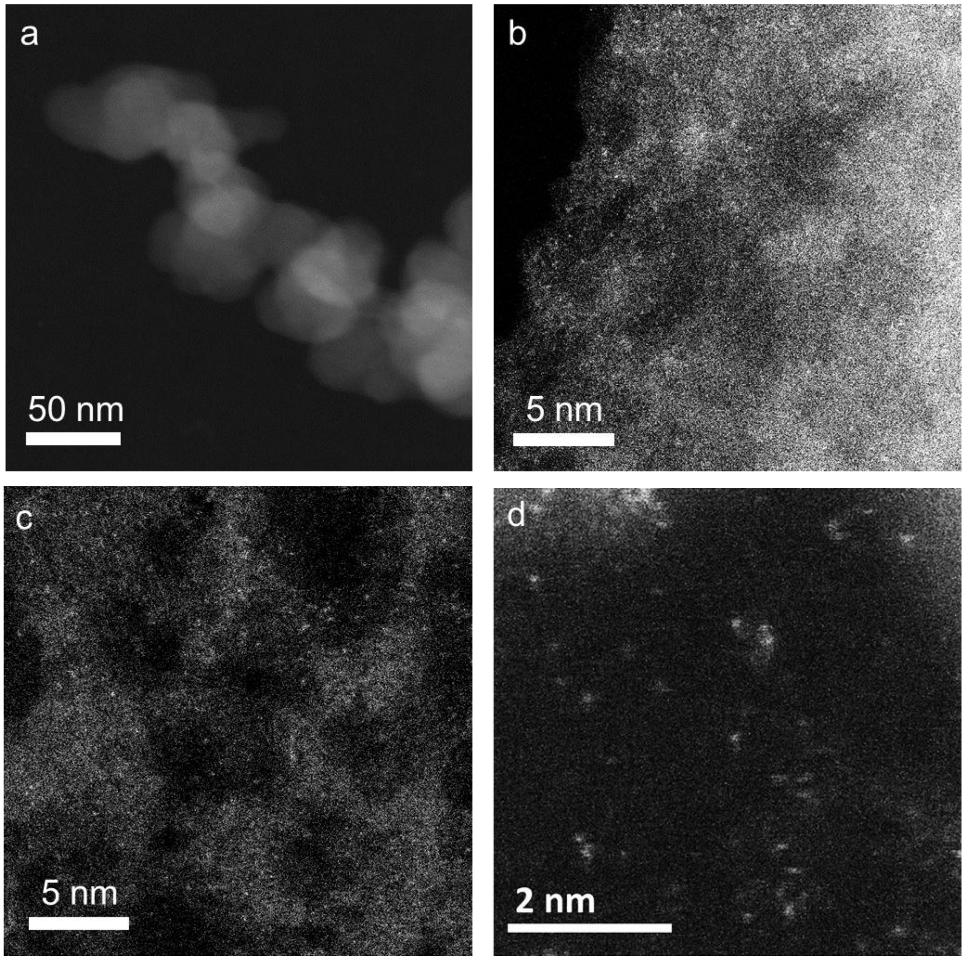

Supplementary Figure 1 (**a**) HAADF-STEM image of Pd_1/CALB-P. (**b-d**) Additional AC-STEM images of Pd_1/CALB-P. Pd_1/CALB-P was adsorbed on graphene oxide and then calcined in air at 250 ^oC for better contrast.

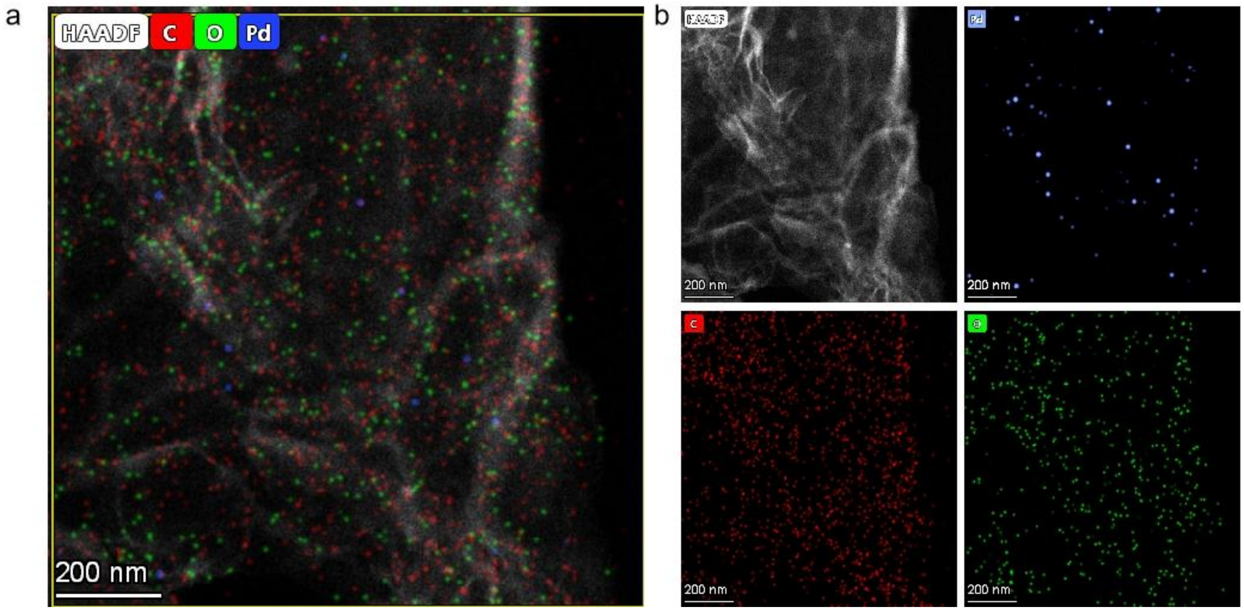

Supplementary Figure 2 Additional energy-dispersive X-ray spectroscopy (EDS) elemental mapping of Pd_1/CALB-P/GO-cal.

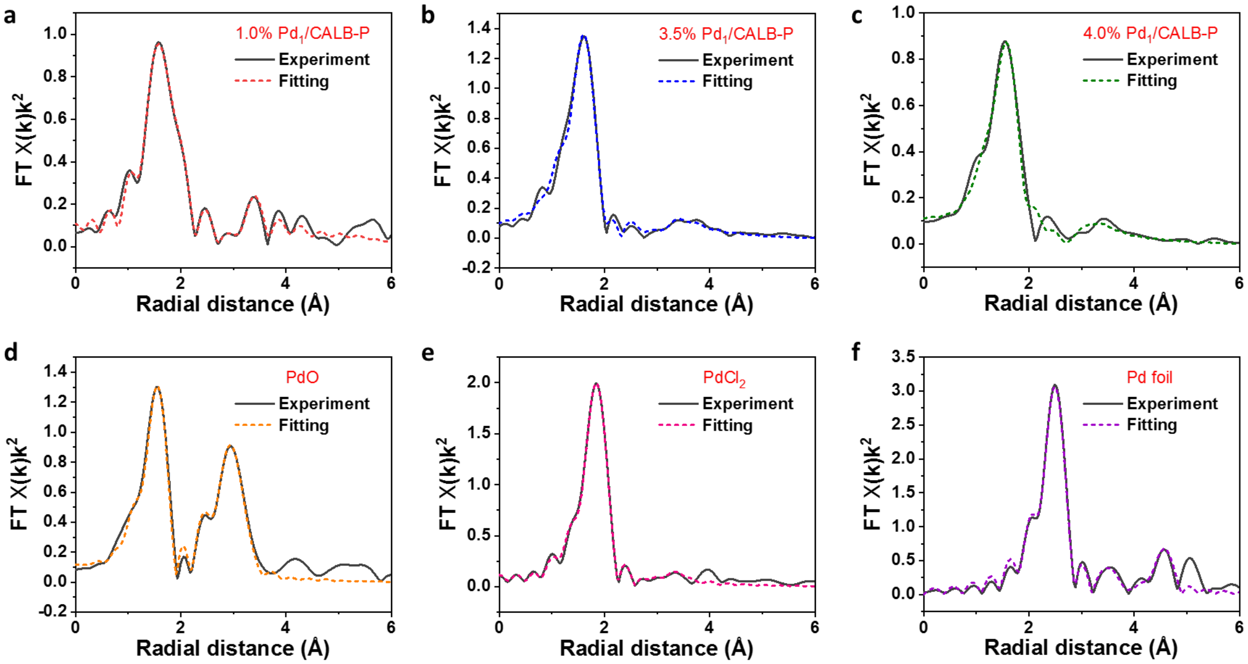

Supplementary Figure 3 Comparison of Fourier transforms and fitting results for EXAFS. (**a**) 1.0% Pd_1/CALB-P; (**b**) 3.5% Pd_1/CALB-P; (**c**) 4.0% Pd_1/CALB-P; (**d**) PdO; (**e**) $PdCl_2$; (**f**) Pd foil. The quantified fitting results were shown in **Supplementary Table 1**.

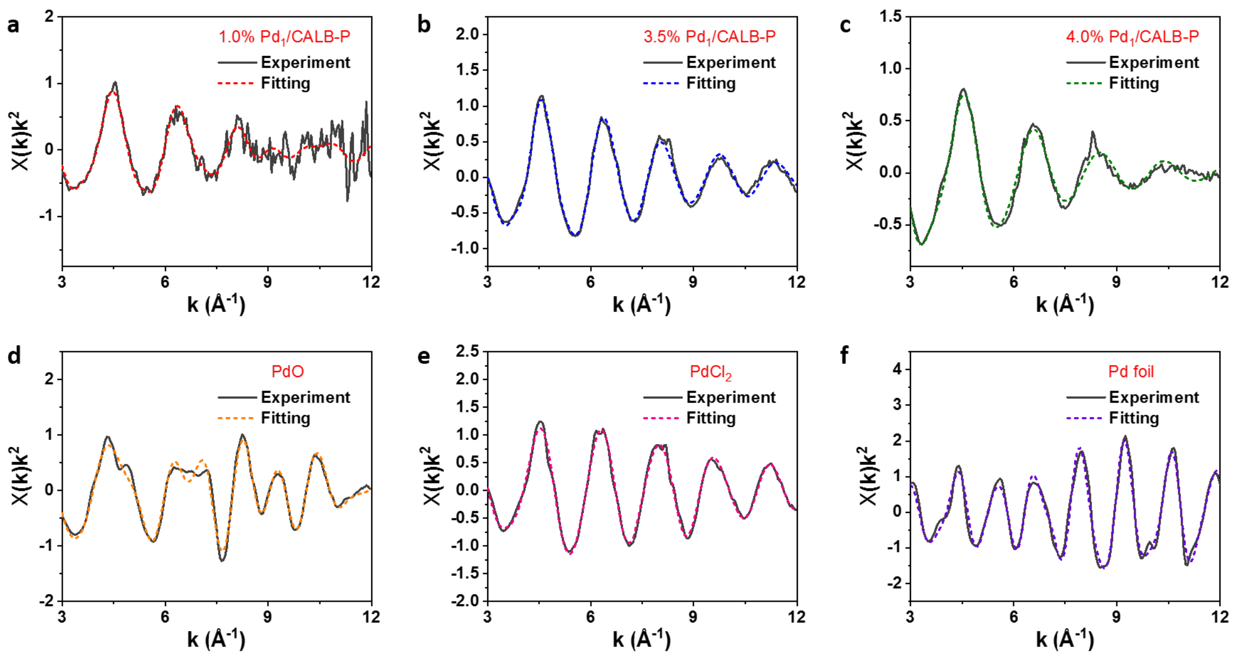

Supplementary Figure 4 The k^2-weighted Pd K-edge EXAFS experimental data and fitting results. (**a**) 1.0% Pd_1/CALB-P; (**b**) 3.5% Pd_1/CALB-P; (**c**) 4.0% Pd_1/CALB-P; (**d**) PdO; (**e**) $PdCl_2$; (**f**) Pd foil. By using Si(311) double crystal monochromator at room temperature, the data collection of 1.0% Pd_1/CALB-P, 3.5% Pd_1/CALB-P and 4.0% Pd_1/CALB-P were carried out in the fluorescent mode with silicon drift fluorescence detector while the PdO, $PdCl_2$ and Pd foil standards were measured in the transmission mode using ion chambers.

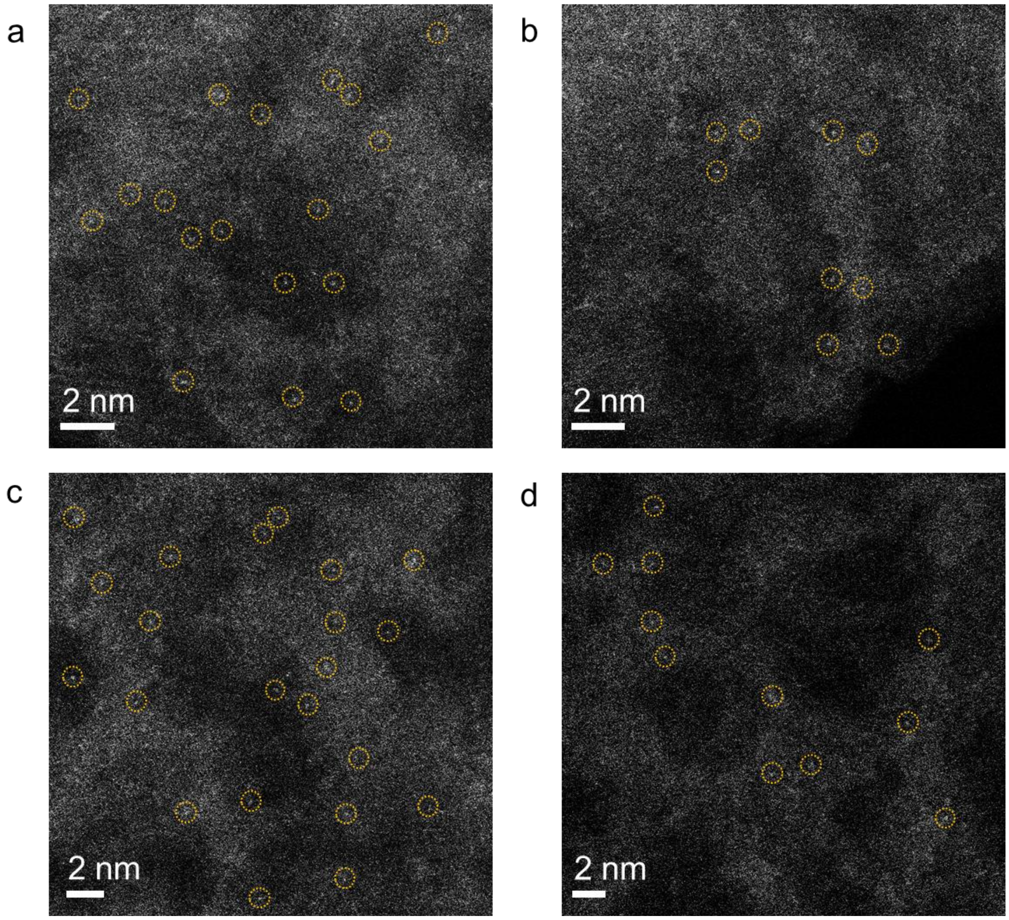

Supplementary Figure 5 AC-STEM images of (**a**) 1.5% Pd_1/ADH-P; (**b**) 0.5% Pd_1/Lac-P; (**c**) 1.9% Pd_1/CAT-P and (**d**) 1.0% Pd_1/GOx-P. The hybrid enzymes were adsorbed on graphene oxide and then calcined in air at 250 ^oC for better contrast.

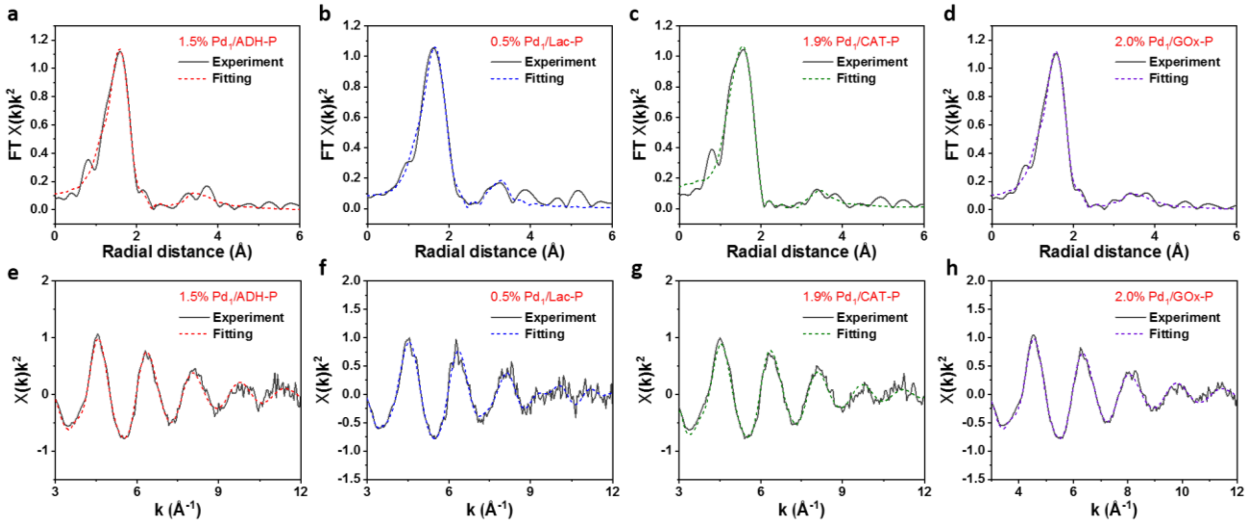

Supplementary Figure 6 Comparison of experimental data and fitting results for EXAFS. (**a**), (**e**) 1.5%Pd_1/ADH -P; (**b**), (**f**) 0.5%Pd_1/Lac-P; (**c**), (**g**) 1.9%Pd_1/CAT-P and (**d**), (**h**) 1.0%Pd_1/GOx-P. The quantified fitting results were shown in **Supplementary Table 1**.

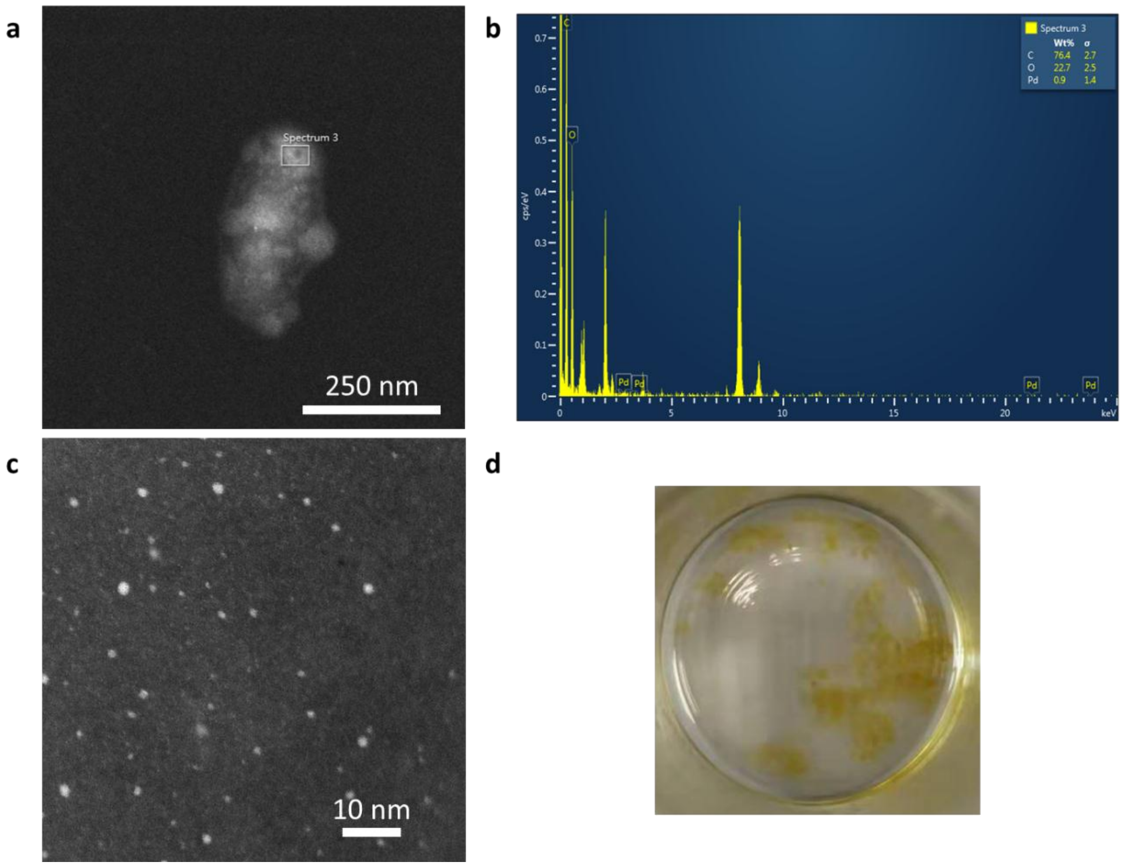

Supplementary Figure 7 (**a**) The HAADF-STEM image of Pd/CALB and (**b**) the corresponding EDS spectrum. (**c**) Magnified HAADF-STEM image of Pd/CALB, showing that atomically dispersed Pd cannot be produced when using the protein alone as the carrier. (**d**) Photo of Pd/CALB in toluene after UV irradiation for 20 minutes, indicating the formation of aggregates.

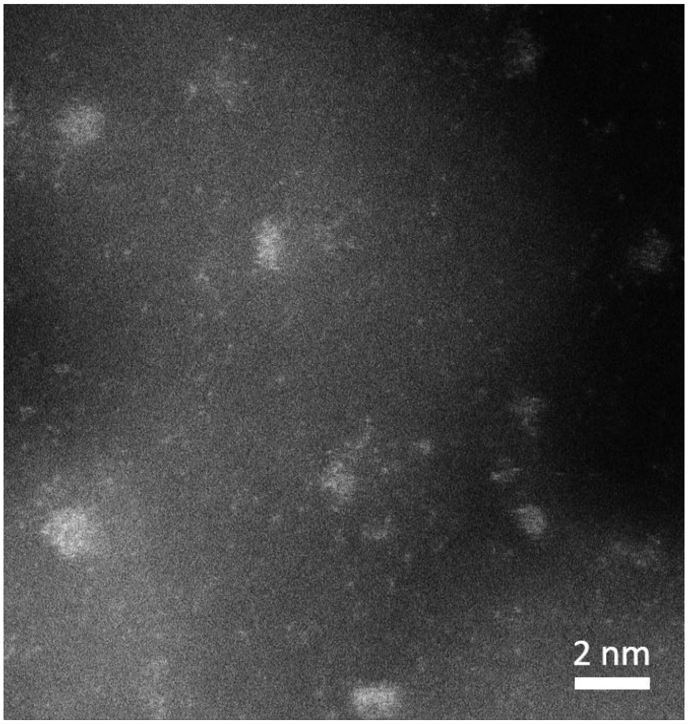

Supplementary Figure 8 HAADF-STEM images of Pd/Pluronic. The sample was adsorbed on GO and then calcined in air at 250 ^{o}C for better contrast.

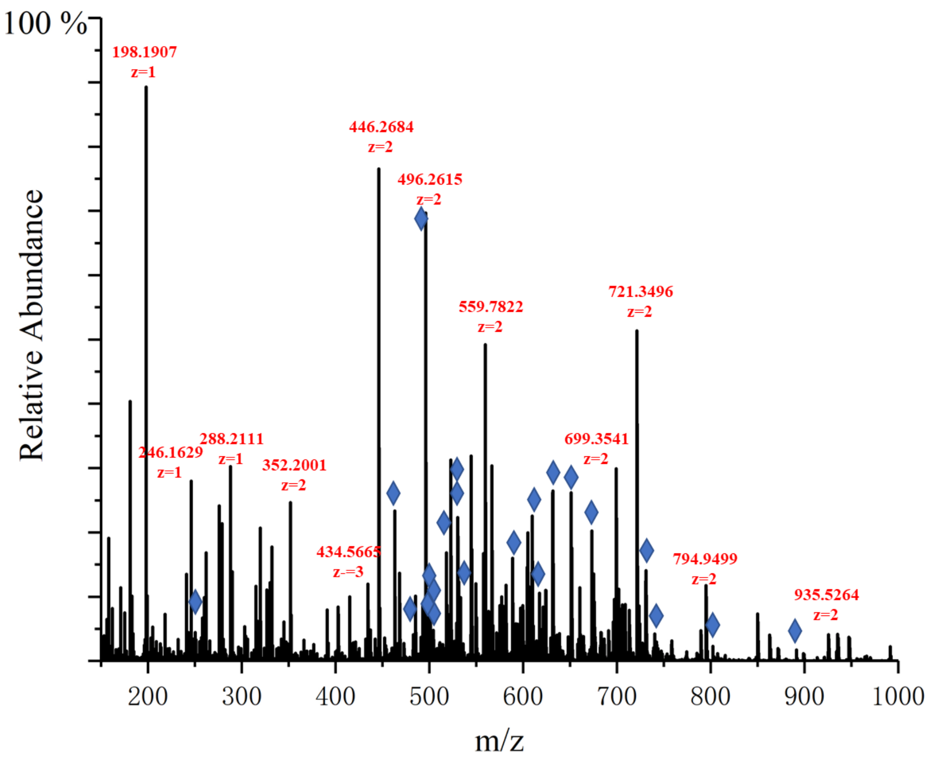

Supplementary Figure 9 Mass spectrum of GOx digested by trypsin in the microdroplet reaction of nanoESI. The results showed that GOx could be digested by trypsin in microdroplets from the tip of nanoESI to the inlet of mass spectrum. 21 peptides could be produced after treating glucose oxidase with trypsin.

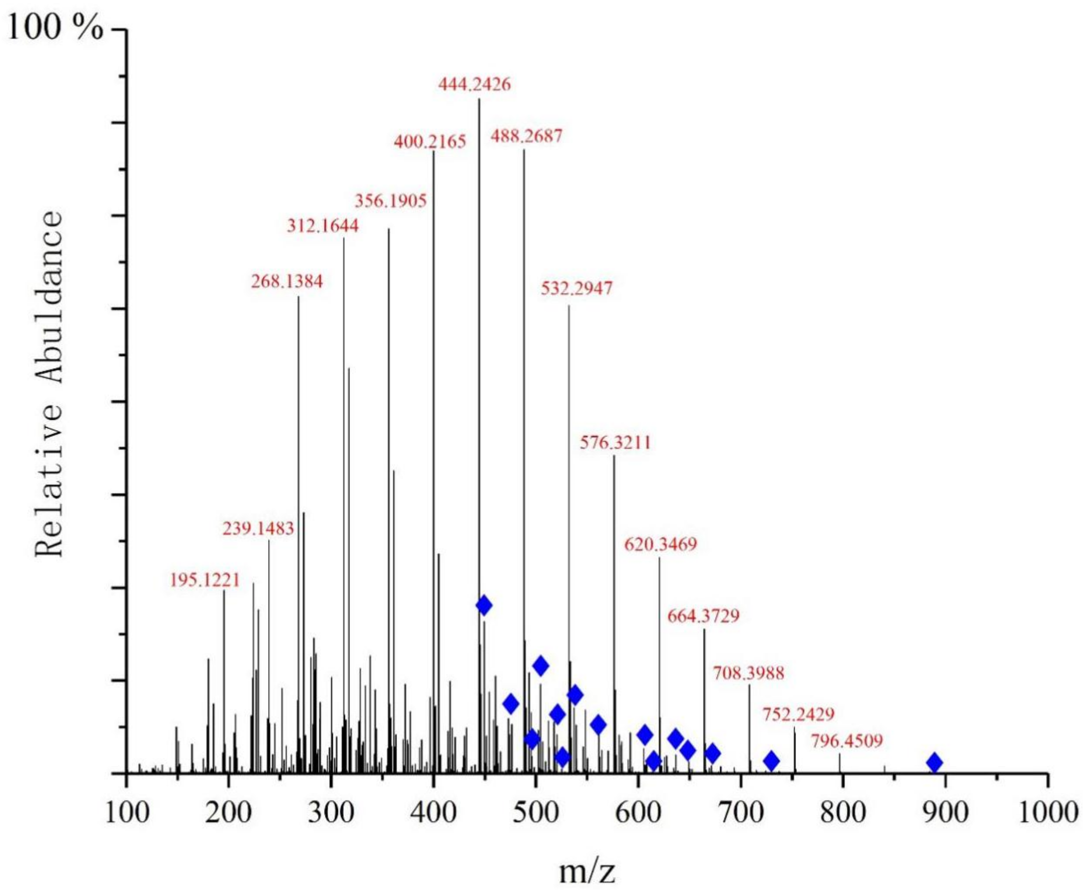

Supplementary Figure 10 Mass spectrum of GOx-Pluronic digested trypsin in the microdroplet reaction of nanoESI. In the mass spectra, there are 13 peptides detected in the digestion of GOx-Pluronic conjugate. The peaks showed mass difference of 44, which was from the ethylene epoxide (C_2H_4O) group of Pluronic, suggesting the successful conjugation of GOx with Pluronic.

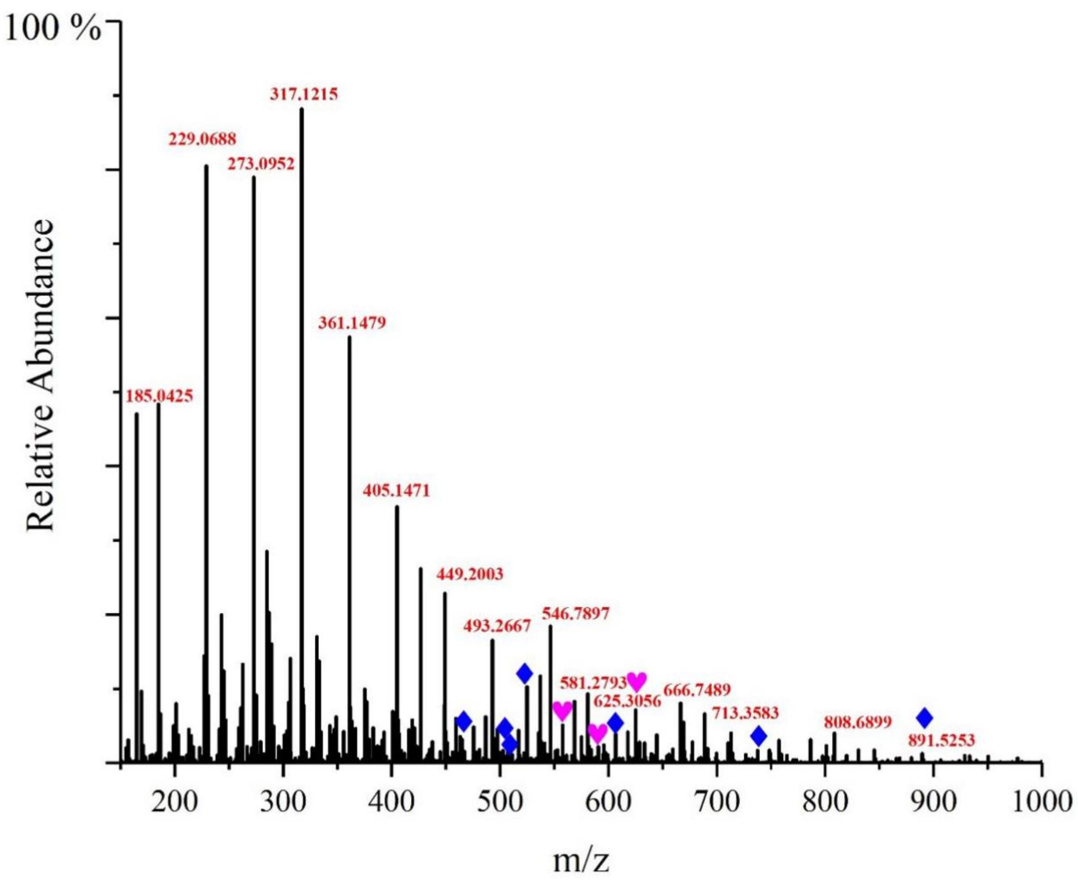

Supplementary Figure 11 Mass spectrum of Pd_1/GOx-Pluronic digested by trypsin in the microdroplet reaction of nanoESI. In the mass spectra, there are 10 peptides detected in the digestion of Pd_1/GOx-Pluronic conjugate. The ethylene epoxide (C_2H_4O) group of Pluronic might resulted in the mass distance of 44 in some peaks. The pink heart squares represent the peptides containing Pd single atoms.

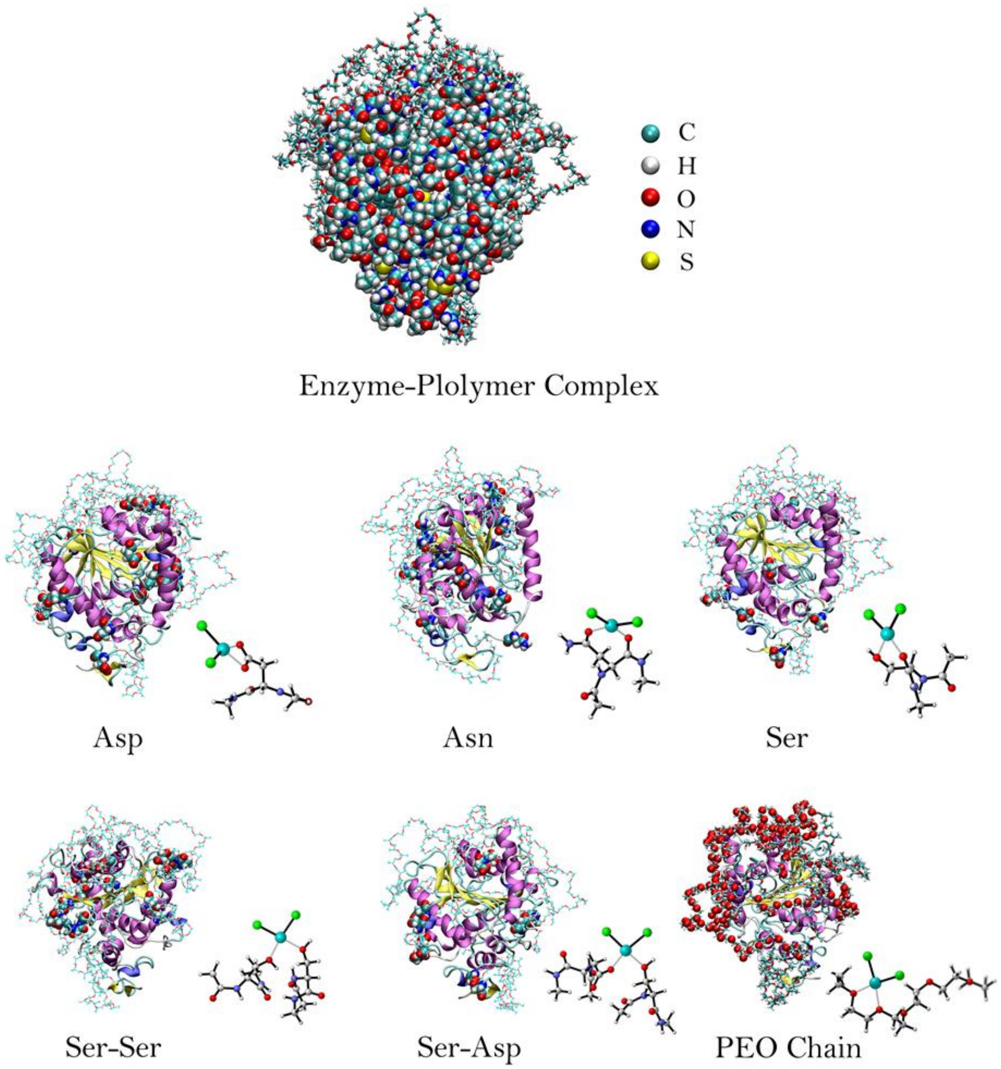

Supplementary Figure 12 Six potential sites anchored $PdCl_2$ and their distributions on the surface of the enzyme-Pluronic conjugate.

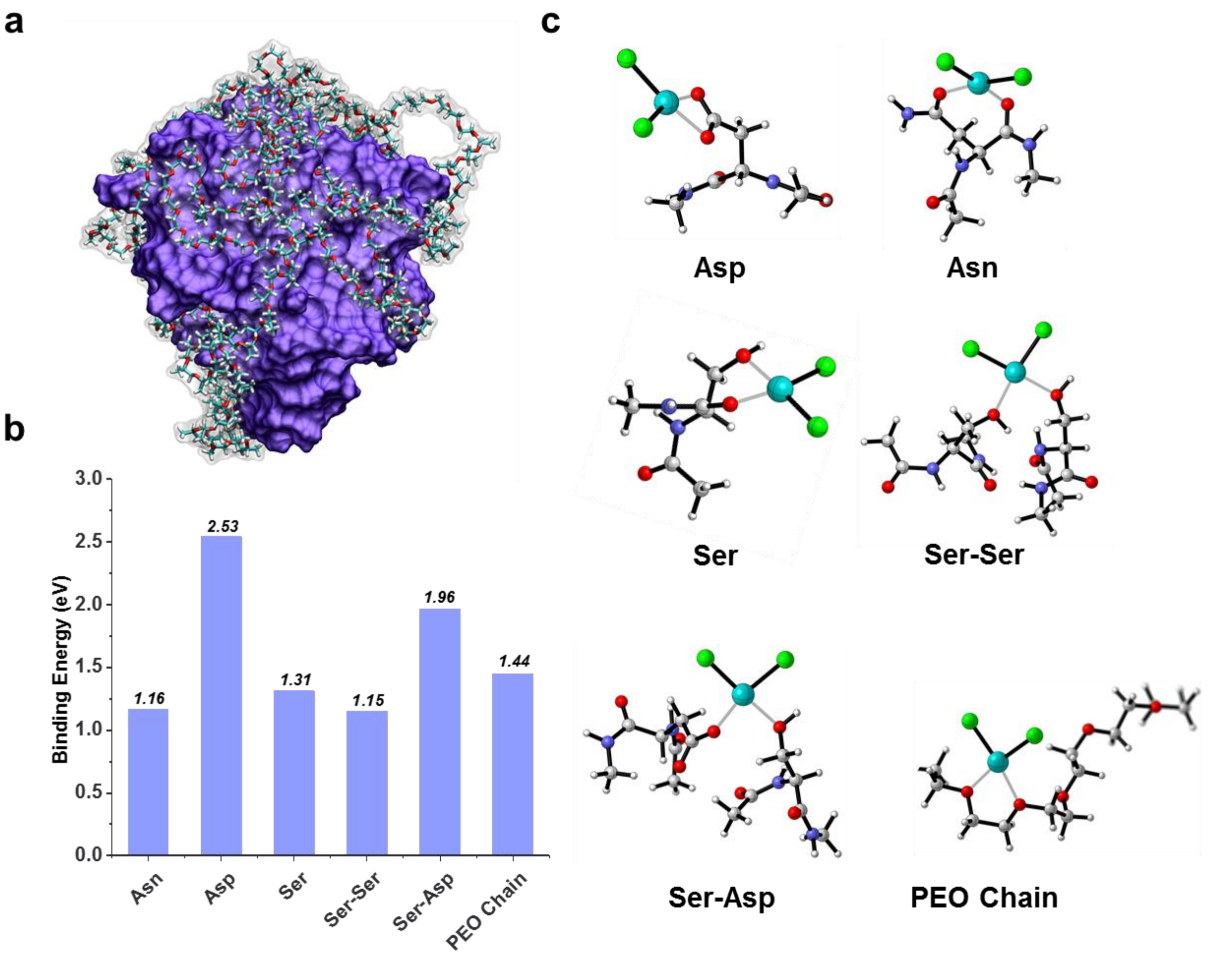

Supplementary Figure 13 (**a**) Conformation of enzyme-Pluronic conjugate. (**b**) Binding energy of six potential sites on which $PdCl_2$ was anchored. (**c**) Optimized structures of six potential sites bound to $PdCl_2$.

a

b

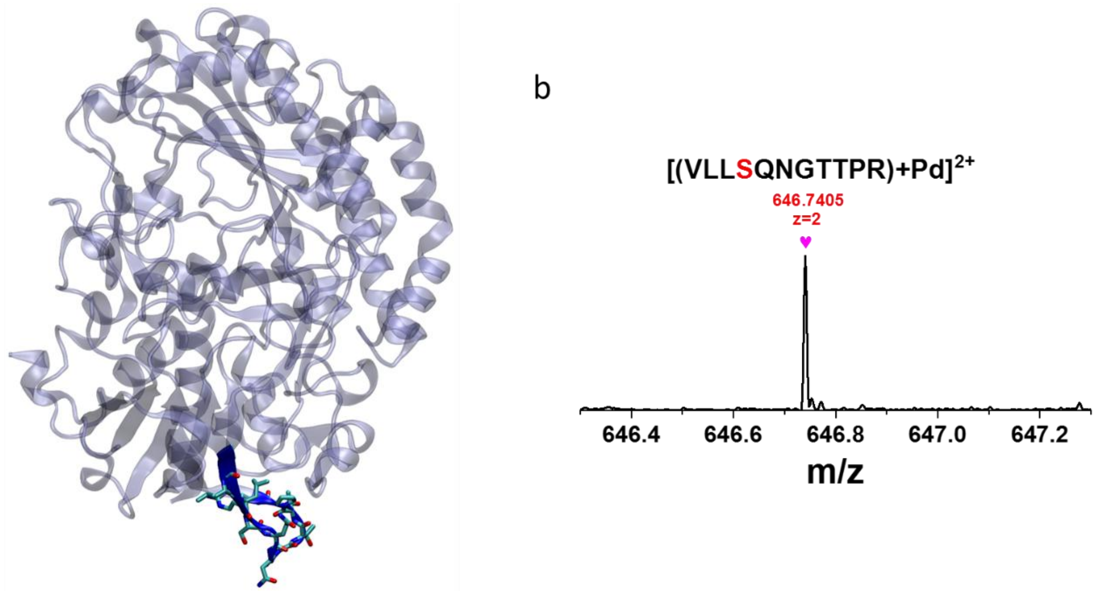

Supplementary Figure 14 Characterization of the possible peptide binding with Pd single atom. The (**a**) configuration and (**b**) mass spectrum of peptide detected from the digestion of Pd_1/GOx-P in the microdroplet reaction by nanoESI. The Ser 278 from VLLSQNGTTPR might be the possible amino acid responsible for coordinating with Pd single atom.

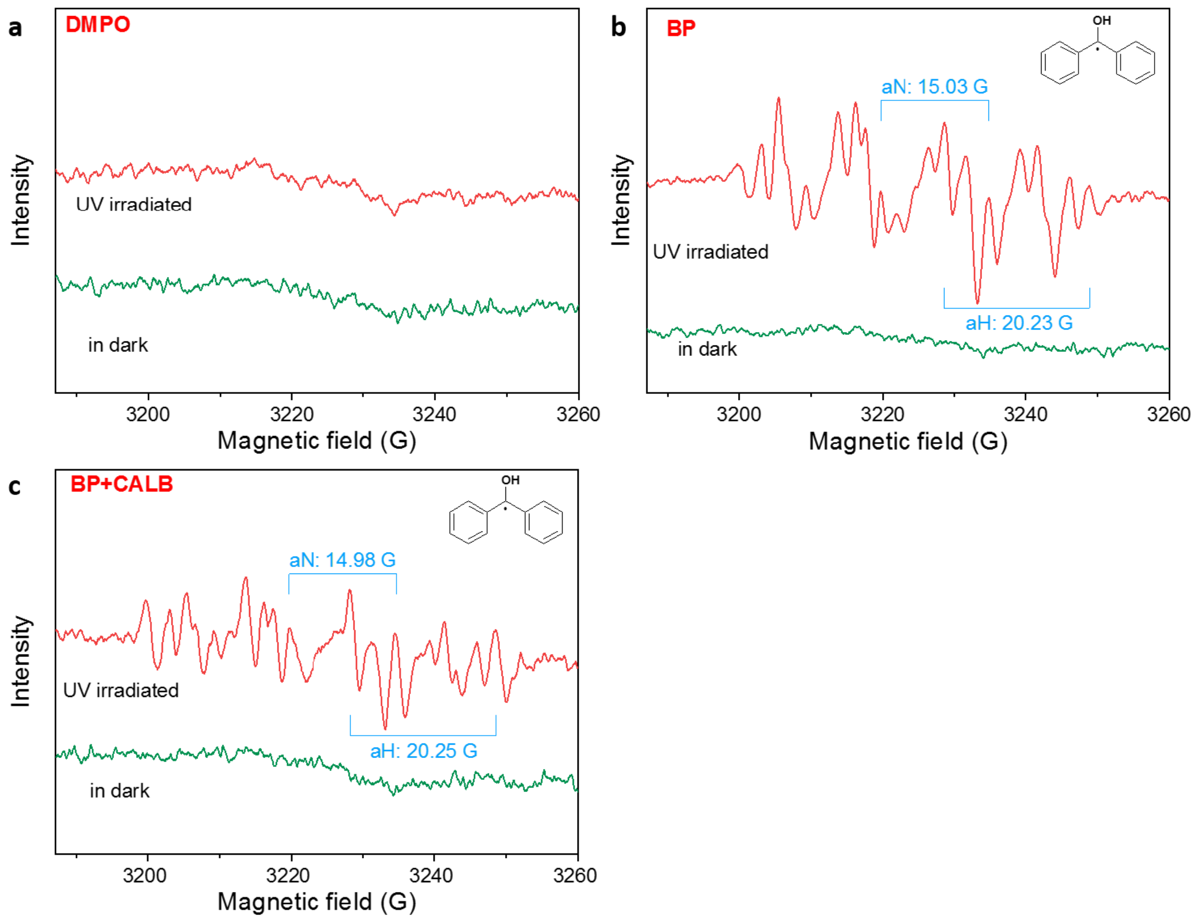

Supplementary Figure 15 The EPR spectra of (**a**) DMPO, (**b**) BP and (**c**) BP+CALB after UV irradiation for 4 min or in dark.

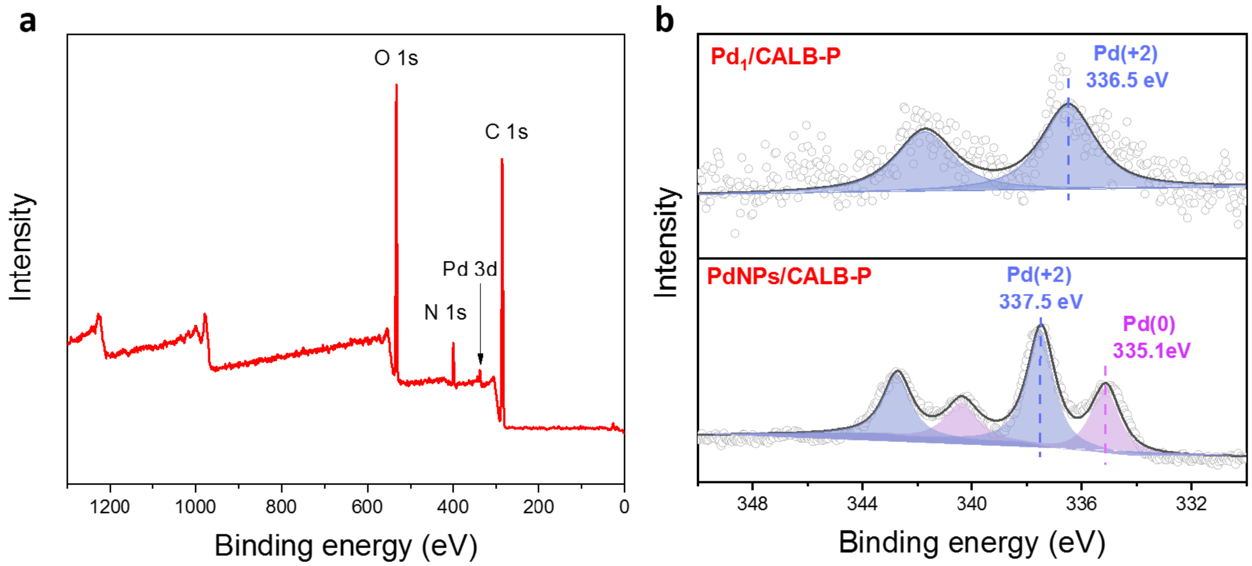

Supplementary Figure 16 XPS analysis of Pd_1/CALB-P. (**a**) The survey spectrum of Pd_1/CALB-P and (**b**) the Pd 3d XPS spectra of Pd_1/CALB-P and PdNPs/CALB-P.

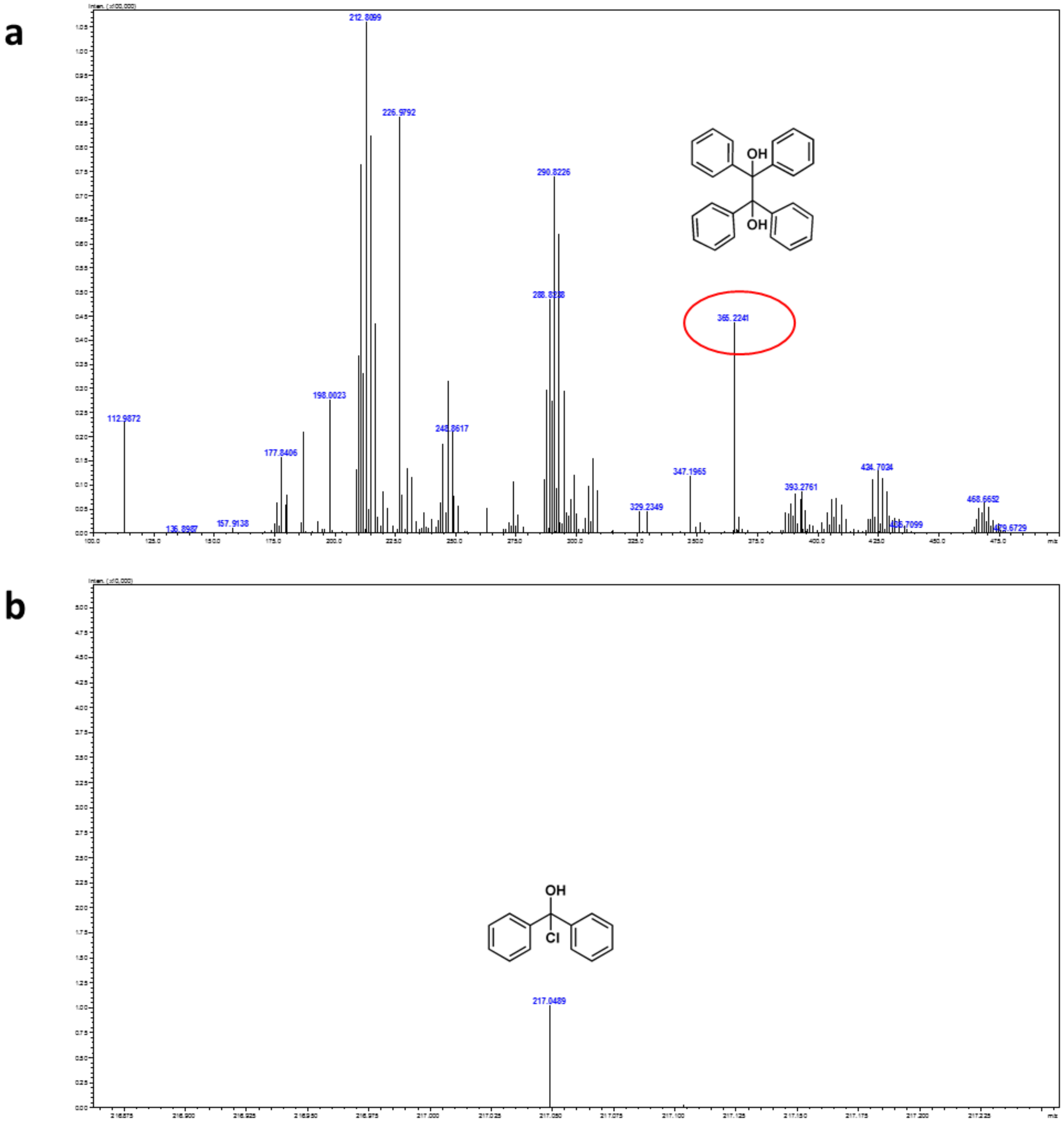

Supplementary Figure 17 ESI-IT-TOF MS spectra for the supernatant separated from the mixture after preparing Pd_1/CALB-P by UV-light irradiation. (**a**) 1,1,2,2-Tetraphenyl-1,2-ethandiol ($[M-H]^-$, $C_{26}H_{22}O_2$, m/z: 365.2241), formed by the coupling of two ketyl radicals; (**b**) 1-Chloro-1,1-diphenyl-methanol ($[M-H]^-$, $C_{13}H_{11}OCl$, m/z: 217.0489). The ESI-IT-TOF-MS was conducted in the negative ion mode.

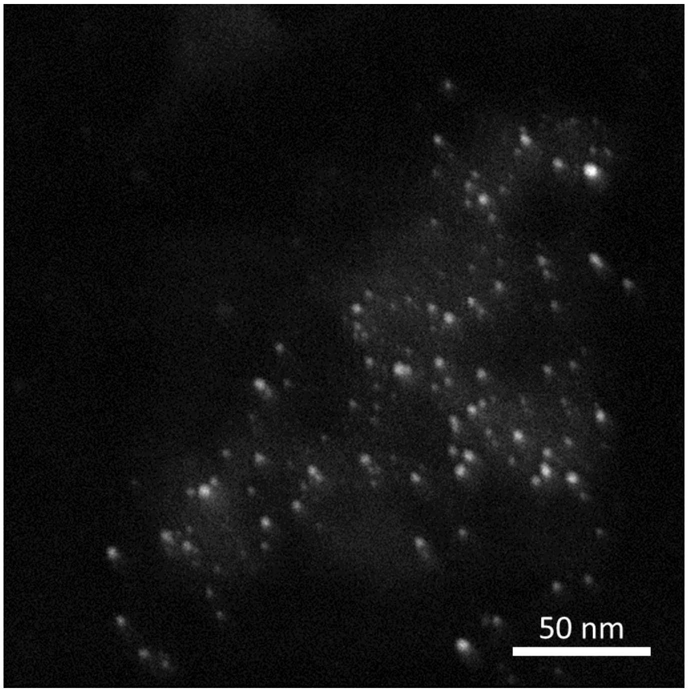

Supplementary Figure 18 HAADF-STEM image of Pd/CALB-P synthesized without using BP as the photoinitiator, suggesting that the radicals formed on the CALB-P conjugates played an important role to stabilize Pd single atoms.

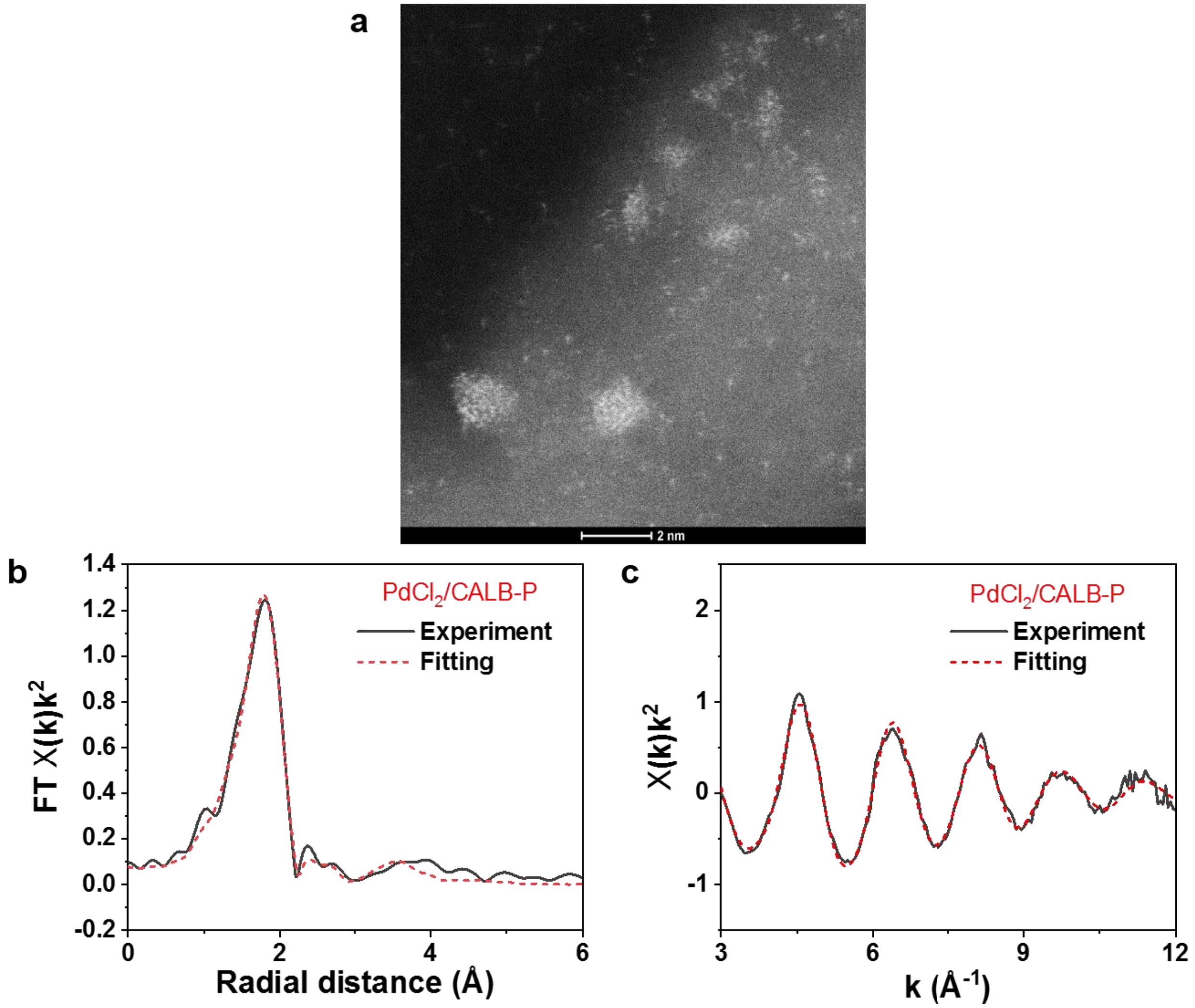

Supplementary Figure 19 Characterization of $PdCl_2$/CALB-P synthesized without UV irradiation. (**a**) AC-STEM image of $PdCl_2$/CALB-P, showing that Pd single atoms cannot be stabilized on CALB-P without the alkyl radicals. $PdCl_2$/CALB-P was adsorbed on TiO_2 and then calcined at 250 ^{o}C for better contrast. (**b**) Comparison of Fourier transforms and fitting results for EXAFS and (**c**) the k^2-weighted Pd K-edge EXAFS experimental data and fitting results for $PdCl_2$/CALB-P.

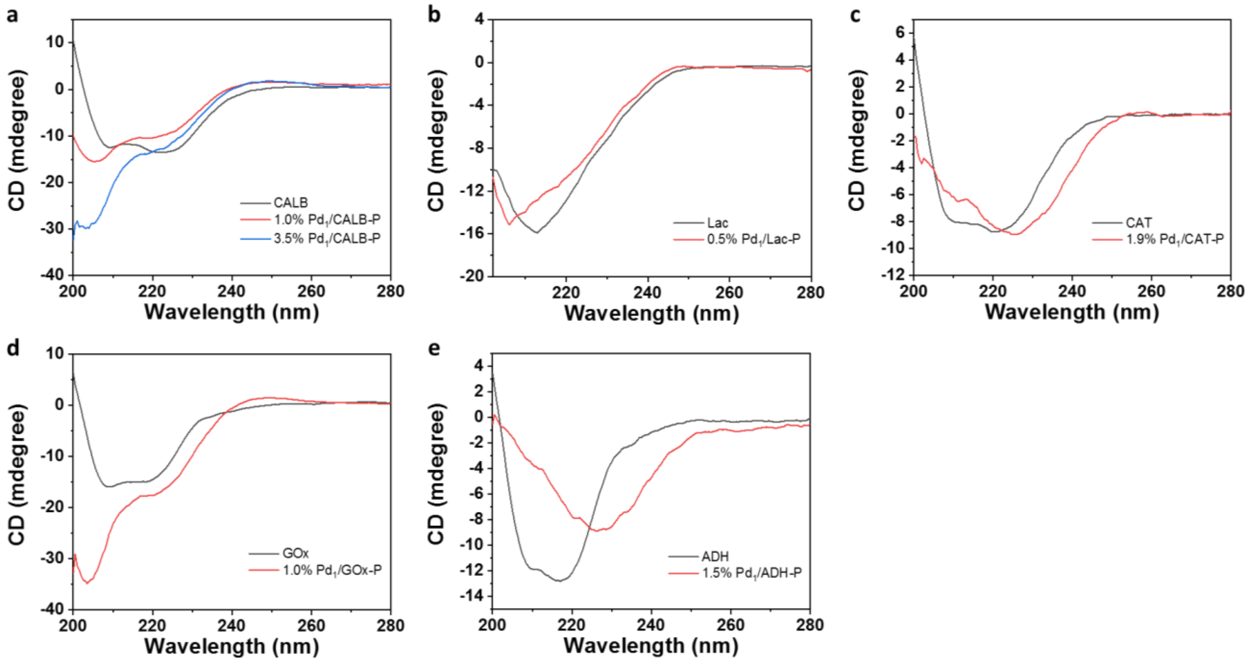

Supplementary Figure 20 Circular dichroism (CD) spectra of (**a**) CALB, 1.0%Pd_1/CALB-P and 3.5%Pd_1/CALB-P; (**b**) ADH and 1.5%Pd_1/ADH-P; (**c**) CAT and 1.9%Pd_1/CAT-P; (**d**) GOx and 1.0%Pd_1/GOx-P; (**e**) Lac and 0.5%Pd_1/Lac-P. The same protein concentration was used for all samples. It suggested that the proteins in the hybrid enzymes remained most of the secondary structures.

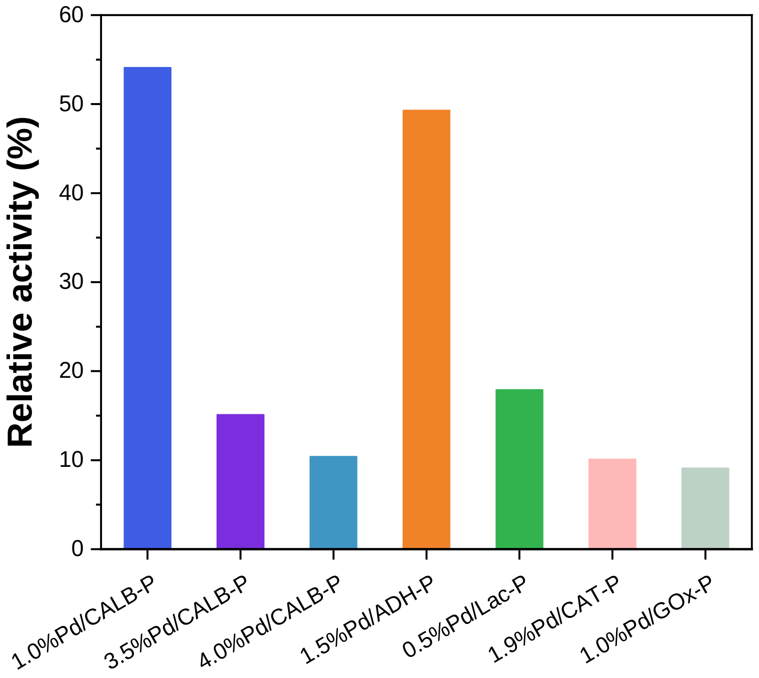

Supplementary Figure 21 Enzymatic activity of the single-atom enzyme-metal complexs. The activities were compared with native enzymes (100%) at the same protein amounts.

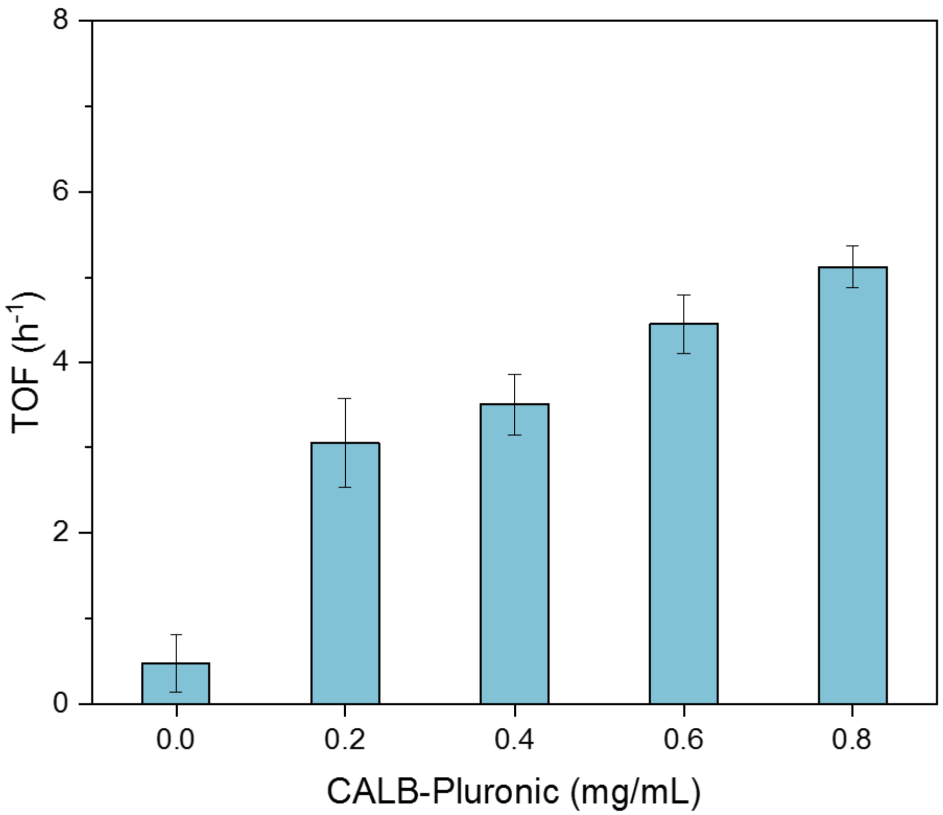

Supplementary Figure 22 TOF of Pd(OAc)$_2$ when using as the catalyst combined with CALB-Pluronic in the cross-coupling of 1-bromohexane and *B*-n-hexyl-9-BBN in H_2O:THF (4:1). Each data point and error bar represent the mean and standard deviation from at least three independent measurements.

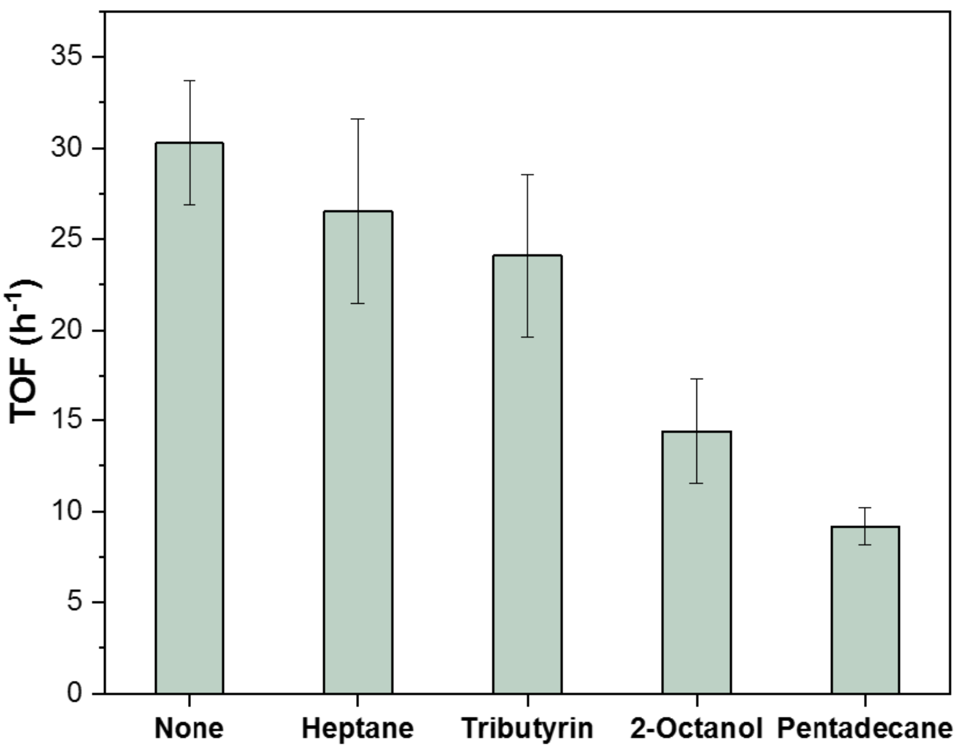

Supplementary Figure 23 TOF of Pd_1/CALB-P in the cross-coupling of 1-bromohexane and *B*-n-hexyl-9-BBN in H_2O:THF (4:1) when heptane, tributyrin, 2-octanol or pentadecane was added as the inhibitor of lipase. The TOF values were calculated with the conversions of 1-bromohexane at the beginning of the reactions (at 5 min). Each data point and error bar represent the mean and standard deviation from at least three independent measurements.

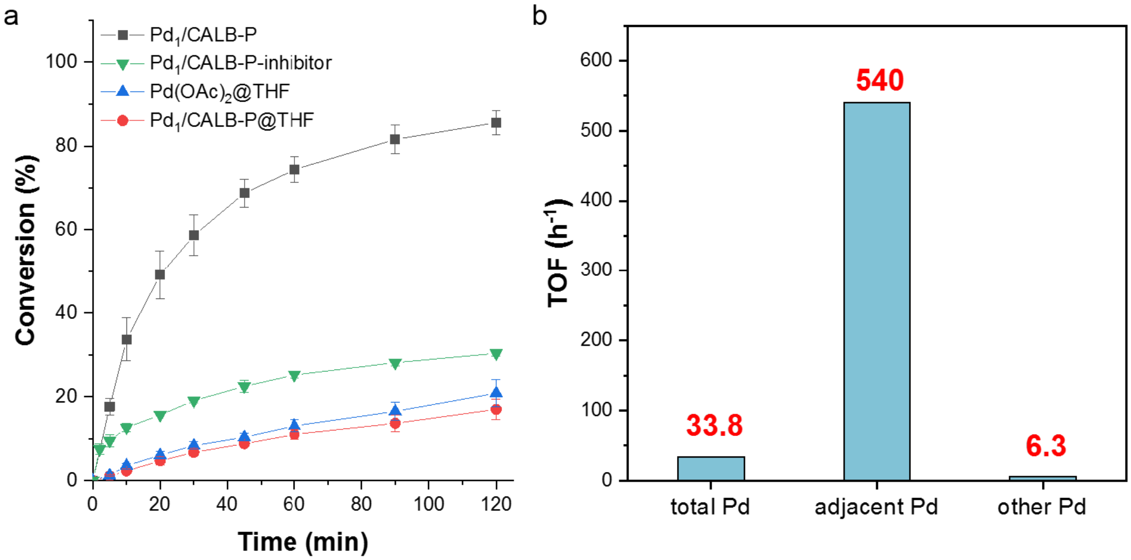

Supplementary Figure 24 Catalytic activity of the enzyme-adjacent Pd single atom. (**a**) Comparison of the catalytic performance of Pd_1/CALB-P in the cross-coupling of 1-bromohexane and *B*-n-hexyl-9-BBN in H_2O:THF (4:1) with and without adding pentadecane as the inhibitor at 2 min. (**b**) TOF of the total Pd single atom (apparent TOF), adjacent Pd (calculated from initial state of 0-2 min of the green curve in the figure a) and other Pd single atoms on the protein surface (calculated from state of 2-5 min of the green curve in the figure a, after the addition of pentadecane to inhibit the adjacent Pd). Each data point and error bar represent the mean and standard deviation from at least three independent measurements.

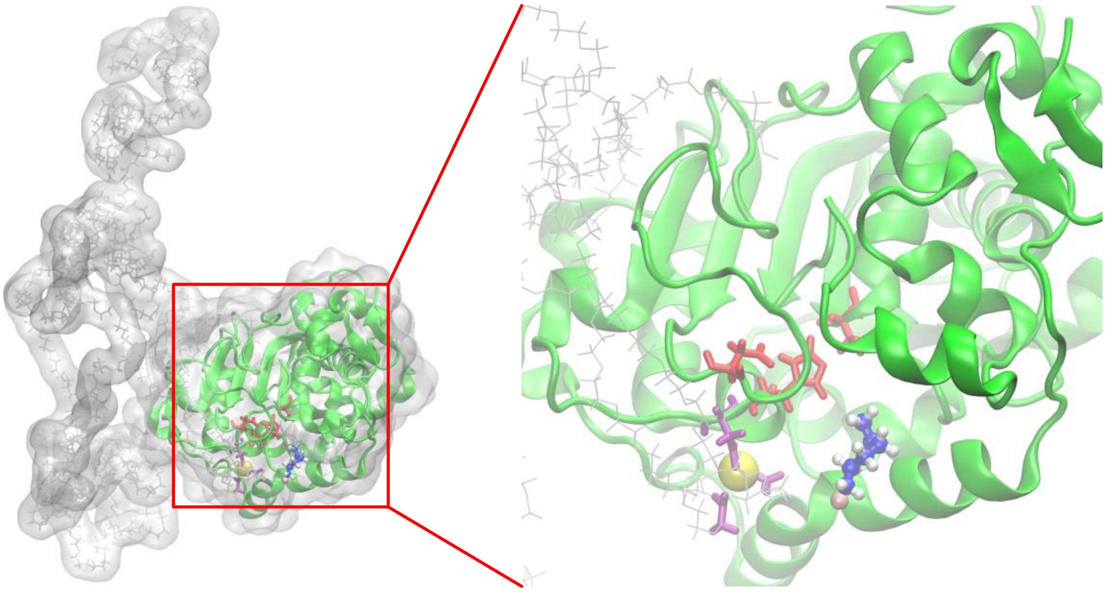

Supplementary Figure 25 All-atom model of Pd_1/CALB-P. The surface is shown by the QuickSurf model. The lipase is shown in green as a cartoon model, where the reported activate site composed of Ser105, Asp187 and His224 is shown in red as a Licorice model. The Pluronic, composed of $(EO)_{100}$-$(PO)_{65}$-$(EO)_{100}$, is shown in grey as a line model. The Pd atom is marked by a yellow bead. The amino acid residues and the EO fragment for the coordination of Pd are shown in purple as a Licorice model. The substrate is shown as a CPK colored ball-and-stick model, except the C atoms are marked in blue. The snapshots are prepared using the VMD software (http://www.ks.uiuc.edu/Research/vmd/) and Rasmol program (http://www.umass.edu/microbio/rasmol/)[1].

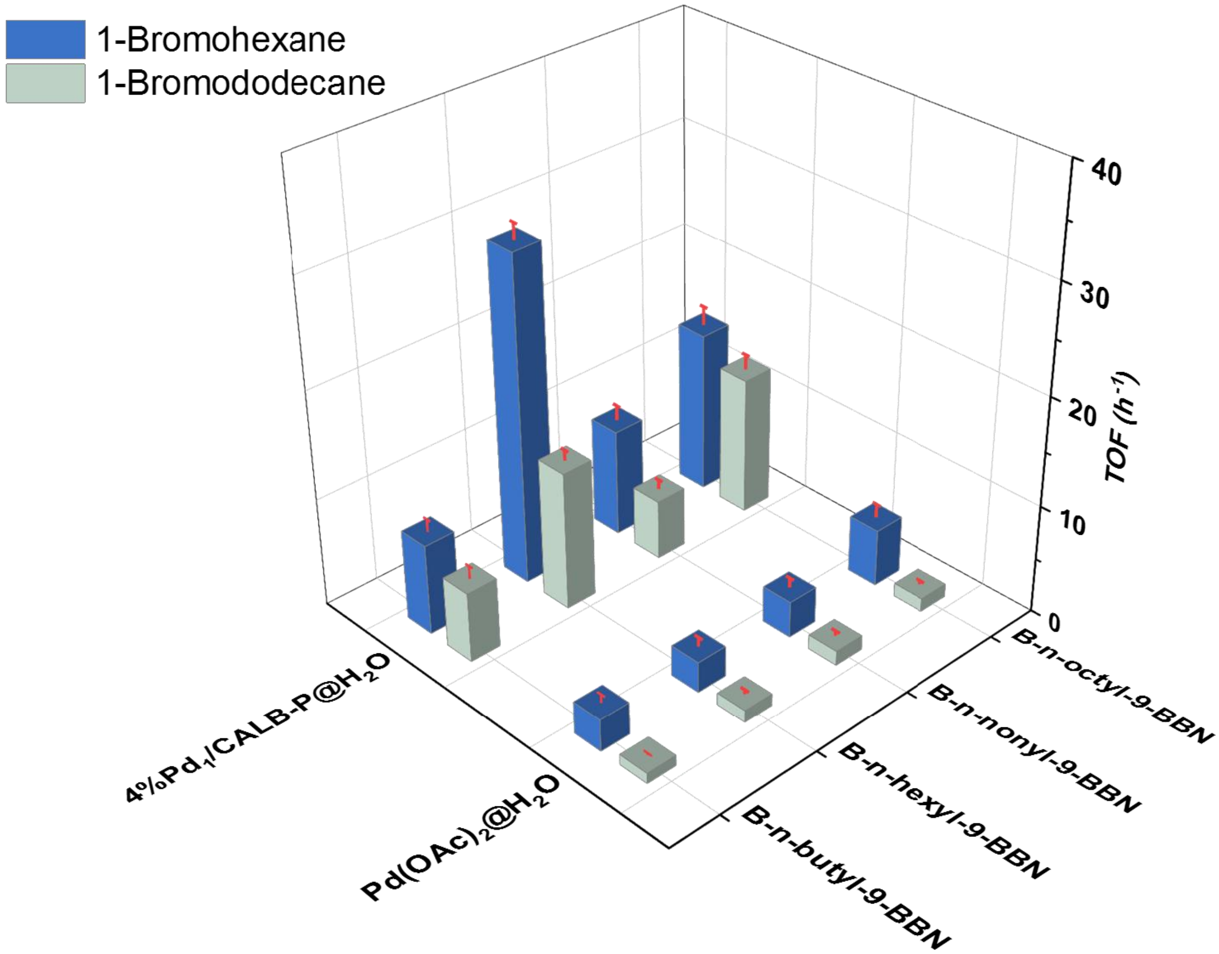

Supplementary Figure 26 TOF of Pd_1/CALB-P and $Pd(OAc)_2$ in the cross-coupling reactions using 1-bromohexane or 1-bromododecane as the electrophile and *B*-n-butyl-9-BBN, *B*-n-butyl-9-BBN, *B*-n-butyl-9-BBN or *B*-n-butyl-9-BBN as the nucleophile. The TOF values were calculated with the conversions of alkyl halide at the beginning of the reactions (at 5 min). Each data point and error bar represent the mean and standard deviation from at least three independent measurements.

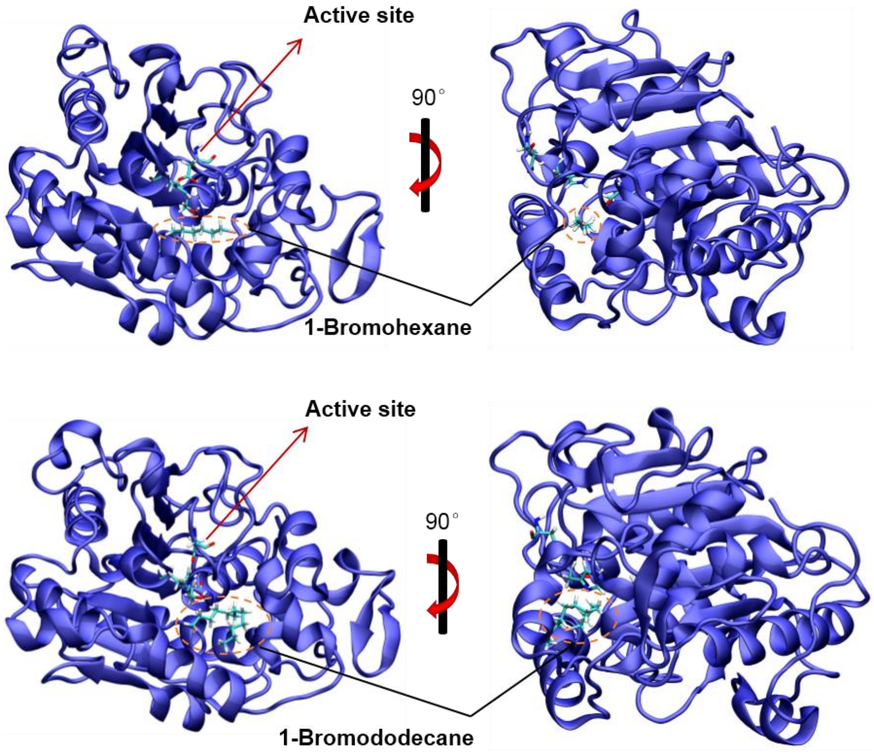

Supplementary Figure 27 The binding of CALB with 1-bromohexane and 1-bromododecane investigated by molecular dynamics simulations. The 1-bromohexane and 1-bromododecan were marked with dotted circles and the active site of CALB was also labeled.

The molecular dynamics simulations were performed using GROMACS 2019.3 [2]. The simulations used the Amber ff99SB-ILDN force field for proteins and the TIP3P model for water[3]. The starting coordinates of the unbound CALB were obtained from the Protein Data Bank (PDB ID: 1TCA)[4]. The 1-bromohexane and 1-bromododecane were built into the free enzyme by using the AutoDock 4.2.6 software[5].The total system was energy minimised by a succession of steepest descent and conjugate gradient methods. Thereafter the solvent was equilibrated for 10 ns at constant temperature (310 K) and pressure (1 bar) (NPT) by restraining the positions of the protein atoms followed by NPT equilibration for another 100 ns without position restrain. The final production runs were carried out at constant temperature (300 K) and pressure (1 bar) (NPT) for 2 ns (200 frames) to calculate binding free energy using MM/GBSA[6]. We used V-rescale thermostat[7] and Parrinello-Rahman barostat[8] to keep the temperature and pressure constant, respectively. The cut-off radius for neighbour searching and non-bonded interactions was taken to be 10 Å and all bonds were constrained using the LINCS algorithm. All the simulation results were visualized by VMD[9].

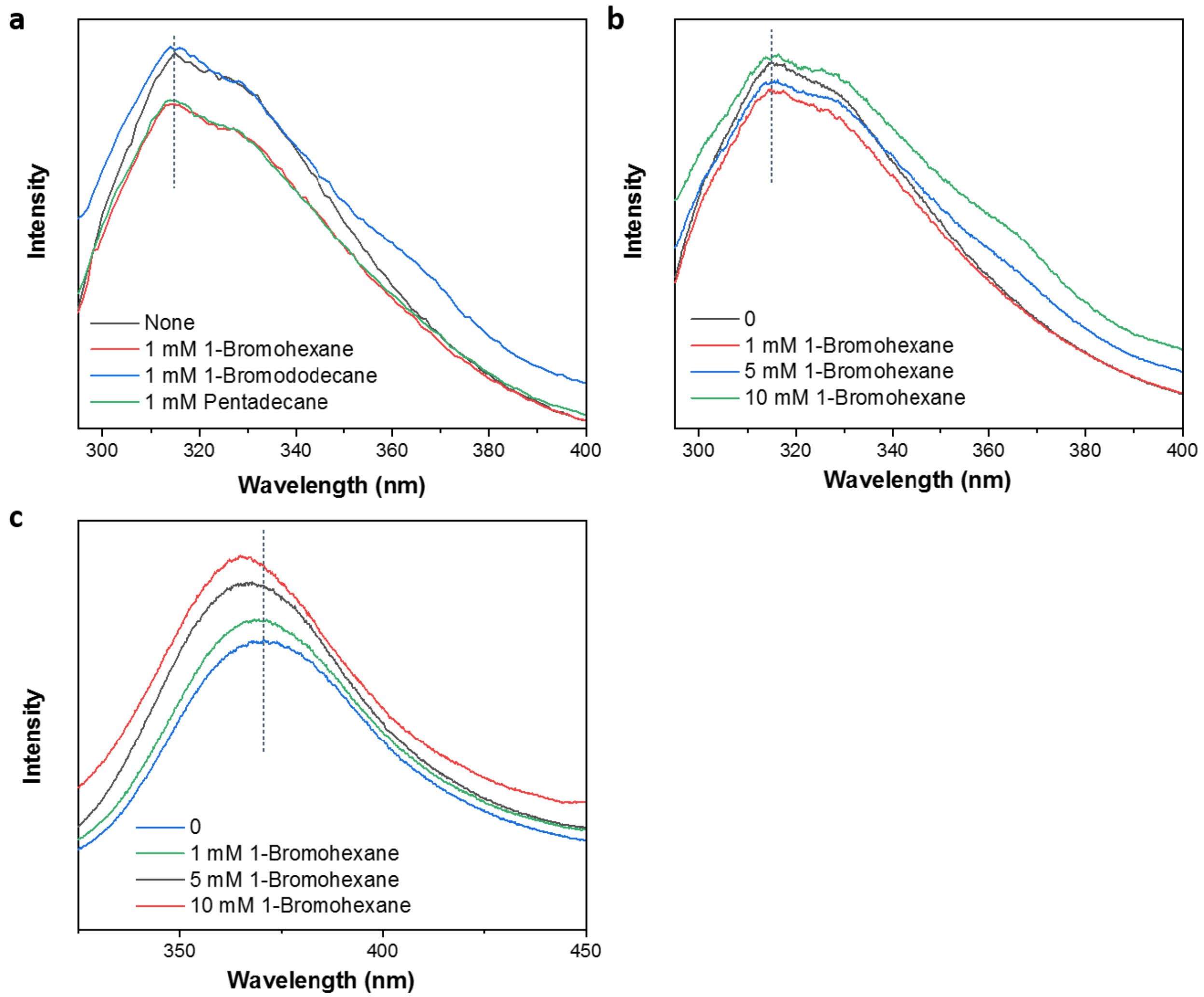

Supplementary Figure 28 Fluorescence spectra of (**a**) Pd_1/CALB-P mixed with different alkyl compounds, (**b**) Pd_1/CALB-P mixed with different concentrations of 1-bromohexane and (**c**) laccase (reference sample) mixed with different concentrations of 1-bromohexane. The same protein concentration was used for all samples. No obvious change in the fluorescence spectrum of Pd_1/CALB-P was detected when mixing with different kinds of alkyl compounds or different concentrations of 1-bromohexane, which was possibly due to that the alkyl compounds were bound at the active site of Pd_1/CALB-P and therefore the microenvironment of the surface tryptophan was not affected by the alkyl compounds. In contrast, for the control sample of native laccase, the maximum fluorescence emission was significantly shifted from 370 nm to 365 nm as the concentration of 1-bromohexane increased from 0 to 10 mM, indicating that the microenvironment of the surface tryptophan became more hydrophobic. This result demonstrated the binding of alkyl compounds at the active site of CALB in the Pd_1/CALB-P catalyst.

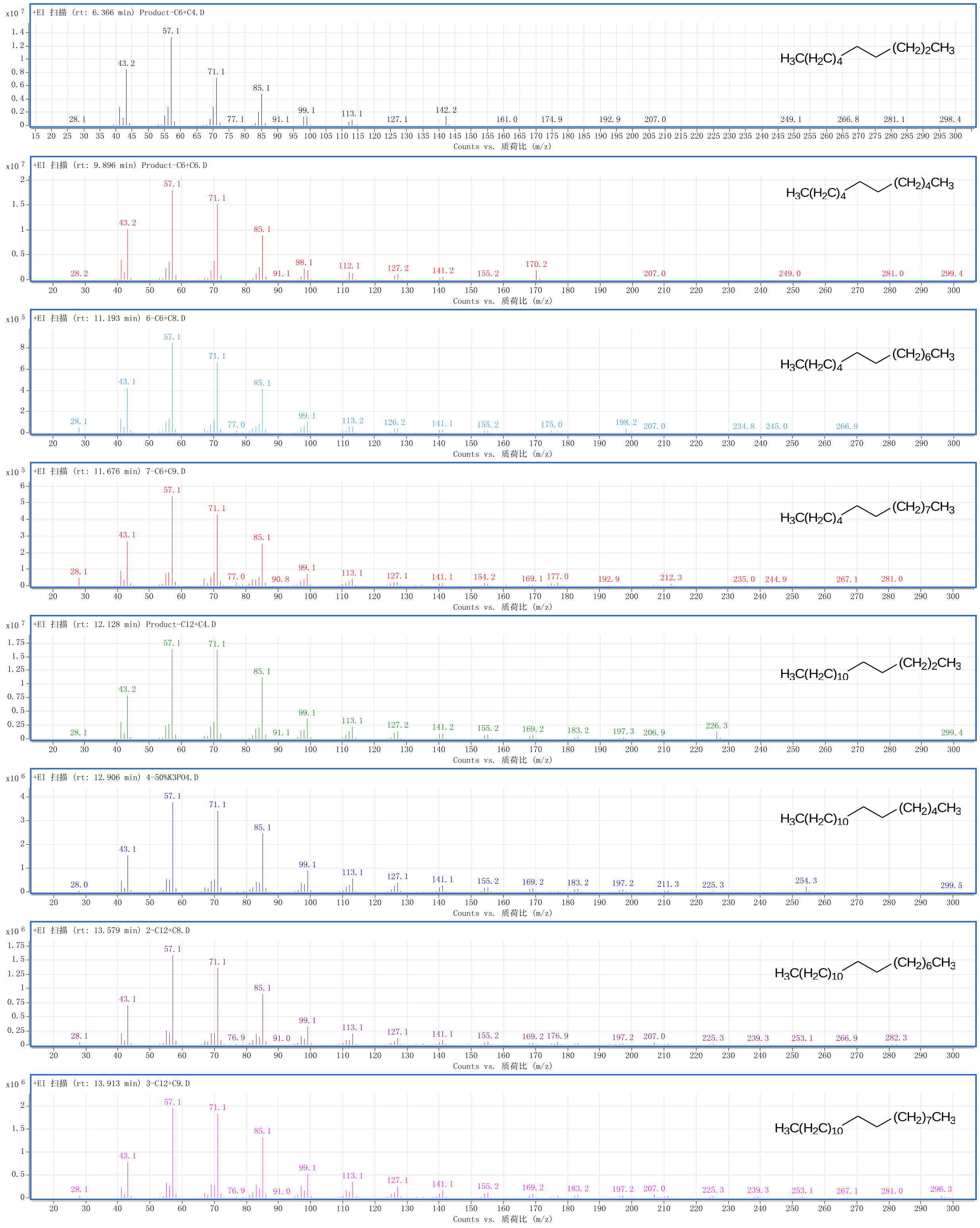

Supplementary Figure 29 Mass spectra of the products in alkyl-alkyl cross-coupling reactions.

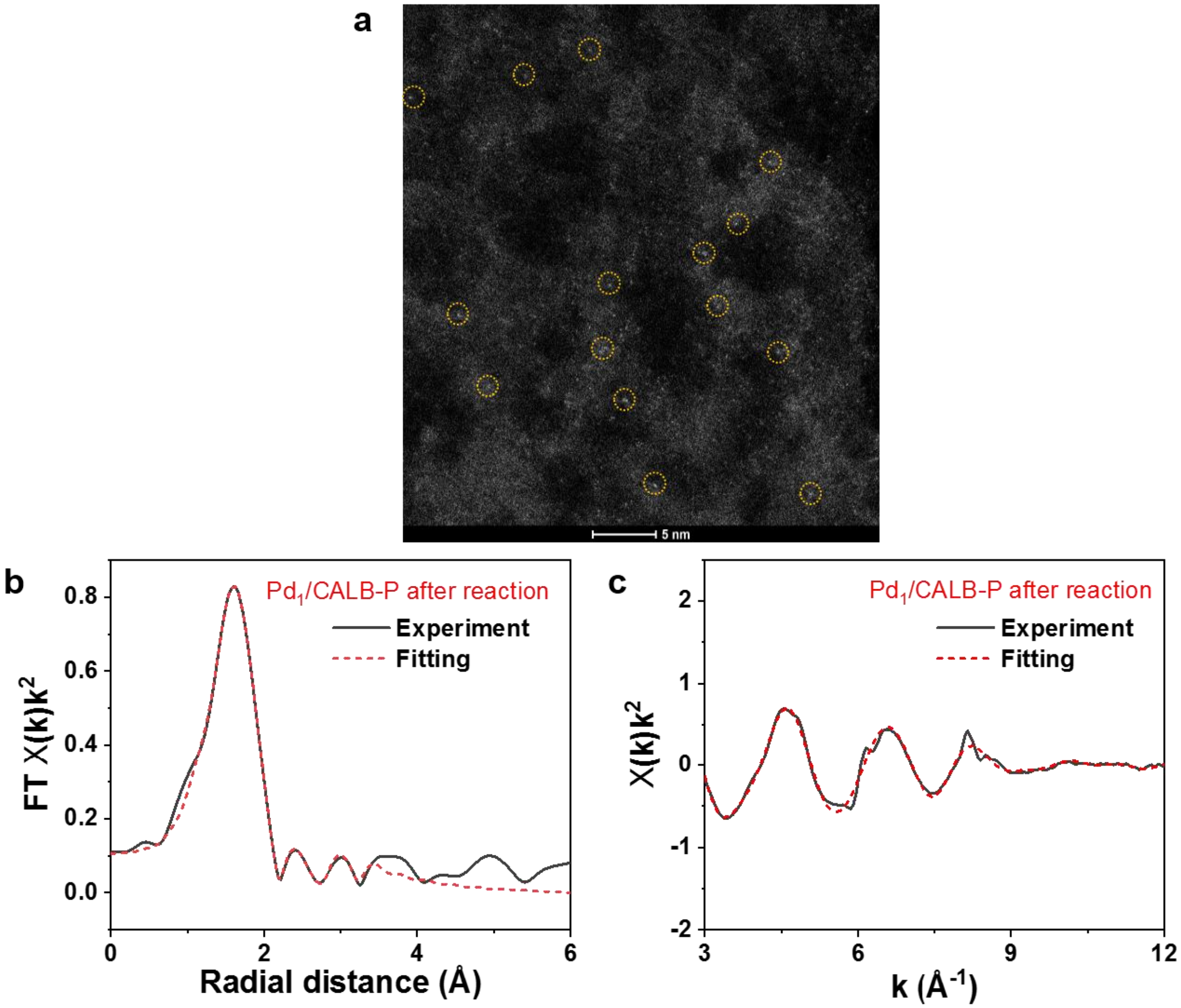

Supplementary Figure 30 Characterization of Pd_1/CALB-P after reactions for 10 cycles. (**a**) AC-STEM image. The sample was adsorbed on GO and then calcined at 250 °C for better contrast. (**b**) Comparison of Fourier transforms and fitting results for EXAFS and (**c**) the k^2-weighted Pd K-edge EXAFS experimental data and fitting results.

Supplementary Table 1 EXAFS parameters of samples.

Sample	Shell	CN	R (Å)	σ^2 (Å^2)	ΔE_0 (eV)
**1.0% Pd_1/CALB-P**	Pd-O/C	4.4	2.097	0.0047	8.8
**3.5% Pd_1/CALB-P**	Pd-O/C	4.7	2.024	0.0038	2.6
**4.0% Pd_1/CALB-P**	Pd-O/C	4.1	2.047	0.0064	7.1
**1.5% Pd_1/ADH-P**	Pd-O/C	5.2	2.020	0.0067	2.5
**0.5% Pd_1/Lac-P**	Pd-O/C	4.8	2.019	0.0098	2.0
**1.9% Pd_1/CAT-P**	Pd-O/C	5.3	2.097	0.0076	9.5
**1.0% Pd_1/GOx-P**	Pd-O/C	4.4	2.089	0.0069	2.2
**$PdCl_2$/CALB-P**	Pd-Cl	1.8	2.324	0.0040	12.3
	Pd-O/C	3.6	2.123	0.0132	7.7
**Pd_1/CALB-P after reaction**	Pd-O/C	3.7	2.054	0.0082	7.4

Note: The analysis was processed by software Artemis, corresponding to the EXAFS spectra in **Supplementary Figure 3, 4, 6, 19 and 30**. CN, coordination number; R, bonding distance; σ^2, Debye-Waller factor; ΔE_0, shift in absorption edge energy.

Supplementary Table 2 Peptides identified by the theoretical simulation digestion of glucose oxidase.

No.	m/z	z	Mass(av)	S-E	Sequence
1	500	1	500.5785	170-174	(R)APNAK(Q)
2	520	1	519.5378	248-252	(R)SDAAR (E)
3	250	2	501.6068	219-223	(R)GVPTK (K)
4	533	1	533.5649	36-40	(K)DVSGR (T)
5	891	1	892.0519	560-567	(R)VYGVQGLR (V)
6	463	2	926.0666	387-394	(K)AHELLNTK (L)
7	473	2	945.1129	456-463	(R)GYVHILDK (D)
8	495	2	992.1457	210-218	(K)ALMSAVEDR (G)
9	502	2	1004.0968	296-304	(K)GNTHNVYAK (H)
10	504	2	1008.1451	210-218	(K)ALMSAVEDR (G)
11	522	2	1045.1903	286-295	(R)AVGVEFGTHK (G)
12	520	2	1119.2489	549-559	(K)EMGGVVDNAAR (V)
13	593	2	1186.3616	275-285	(K)VLLSQNGTTPR (A)
14	605	2	1210.2906	199-209	(R)DTGDDYSPIVK (A)
15	630	1	629.7820	219-224	(R)GVPTKK (D)
16	651	2	1302.4381	395-405	(K)LEQWAEEAVAR (G)
17	673	2	1345.5013	23-35	(R)SNGIEASLLTDPK (D)
18	728	2	1456.6212	593-605	(K)ISDAILEDYASMQ (-)
19	745	2	1490.7291	210-223	(K)ALMSAVEDRGVPTK(K)
20	802	2	1602.9049	210-224	(K)ALMSAVEDRGVPTKK (D)
21	617	3	1851.1128	41-59	(R)TVDYIIAGGGLTGLTTAAR (L)

Supplementary Table 3 The match result (red) for the whole theoretical simulation digestion and mass spectra of glucose oxidase by trypsin.

**1**	MQTLLVSSLV	VSLAAALPHY	IRSNGIEASL	LTDPKDVSGR	TVDYIIAGGG	LTGLTTAARL	TENPNISVLV	IESGSYESDR	
**81**	GPIIEDLNAY	GDIFGSSVDH	AYETVELATN	NQTALIRSGN	GLGGSTLVNG	GTWTRPHKAQ	VDSWETVFGN	EGWNWDNVAA	
**161**	YSLQAERARA	PNAKQIAAGH	YFNASCHGVN	GTVHAGPRDT	GDDYSPIVKA	LMSAVEDRGV	PTKKDFGCGD	PHGVSMFPNT	
**241**	LHEDQVRSDA	AREWLLPNYQ	RPNLQVLTGQ	YVGKVLLSQN	GTTPRAVGVE	FGTHKGNTHN	VYAKHEVLLA	AGSAVSPTIL	
**321**	EYSGIGMKSI	LEPLGIDTVV	DLPVGLNLQD	QTTATVRSRI	TSAGAGQGQA	AWFATFNETF	GDYSEKAHEL	LNTKLEQWAE	
**401**	EAVARGGFHN	TTALLIQYEN	YRDWIVNHNV	AYSELFLDTA	GVASFDVWDL	LPFTRGYVHI	LDKDPYLHHF	AYDPQYFLNE	
**481**	LDLLGQAAAT	QLARNISNSG	AMQTYFAGET	IPGDNLAYDA	DLSAWTEYIP	YHFRPNYHGV	GTCSMMPKEM	GGVVDNAARV	
**561**	YGVQGLRVID	GSIPPTQMSS	HVMTVFYAMA	LKISDAILED	YASMQ				

Supplementary Table 4 Peptides identified by the theoretical simulation digestion of GOx (black) and GOx-Pluronic (blue).

No.	m/z	z	Mass(av)	S-E	Sequence
1	500	1	500.5785	170-174	(R)APNAK(Q)
2	520	1	519.5378	248-252	(R)SDAAR (E)
3	250	2	501.6068	219-223	(R)GVPTK (K)
4	533	1	533.5649	36-40	(K)DVSGR (T)
5	891	1	892.0519	560-567	(R)VYGVQGLR (V)
6	463	2	926.0666	387-394	(K)AHELLNTK (L)
7	473	2	945.1129	456-463	(R)GYVHILDK (D)
8	495	2	992.1457	210-218	(K)ALMSAVEDR (G)
9	502	2	1004.0968	296-304	(K)GNTHNVYAK (H)
10	504	2	1008.1451	210-218	(K)ALMSAVEDR (G)
11	522	2	1045.1903	286-295	(R)AVGVEFGTHK (G)
12	560	2	1119.2489	549-559	(K)EMGGVVDNAAR (V)
13	593	2	1186.3616	275-285	(K)VLLSQNGTTPR (A)
14	605	2	1210.2906	199-209	(R)DTGDDYSPIVK (A)
15	630	1	629.7820	219-224	(R)GVPTKK (D)
16	651	2	1302.4381	395-405	(K)LEQWAEEAVAR (G)
17	673	2	1345.5013	23-35	(R)SNGIEASLLTDPK (D)
18	728	2	1456.6212	593-605	(K)ISDAILEDYASMQ (-)
19	745	2	1490.7291	210-223	(K)ALMSAVEDRGVPTK(K)
20	802	2	1602.9049	210-224	(K)ALMSAVEDRGVPTKK (D)
21	617	3	1851.1128	41-59	(R)TVDYIIAGGGLTGLTTAAR (L)

Supplementary Table 5 Peptides identified by the theoretical simulation digestion of GOx (black) and Pd_1/GOx-Pluronic (green). The segments representing peptides containing Pd atoms were highlight in violet.

No.	m/z	z	Mass(av)	S-E	Sequence
1	500	1	500.5785	170-174	(R)APNAK(Q)
2	626	1	519.5378	248-252	(R)SDAAR (E)-Pd
3	250	2	501.6068	219-223	(R)GVPTK (K)
4	533	1	533.5649	36-40	(K)DVSGR (T)
5	891	1	892.0519	560-567	(R)VYGVQGLR (V)
6	463	2	926.0666	387-394	(K)AHELLNTK (L)
7	473	2	945.1129	456-463	(R)GYVHILDK (D)
8	495	2	992.1457	210-218	(K)ALMSAVEDR (G)
9	502	2	1004.0968	296-304	(K)GNTHNVYAK (H)
10	504	2	1008.1451	210-218	(K)ALMSAVEDR (G)
11	522	2	1045.1903	286-295	(R)AVGVEFGTHK (G)
12	612	2	1119.2489	549-559	(K)EMGGVVDNAAR (V)-Pd
13	646	2	1186.3616	275-285	(K)VLLSQNGTTPR (A)-Pd
14	605	2	1210.2906	199-209	(R)DTGDDYSPIVK (A)
15	630	1	629.7820	219-224	(R)GVPTKK (D)
16	651	2	1302.4381	395-405	(K)LEQWAEEAVAR (G)
17	673	2	1345.5013	23-35	(R)SNGIEASLLTDPK (D)
18	728	2	1456.6212	593-605	(K)ISDAILEDYASMQ (-)
19	745	2	1490.7291	210-223	(K)ALMSAVEDRGVPTK(K)
20	802	2	1602.9049	210-224	(K)ALMSAVEDRGVPTKK (D)
21	617	3	1851.1128	41-59	(R)TVDYIIAGGGLTGLTTAAR (L)

Supplementary Table 6 Secondary structural elements from the CD spectra of native and hybrid enzymes.

Enzyme	α-Helix (%)	β-Sheet (%)	β-Turns (%)	Unordered (%)
CALB	84.8	6.9	2.2	6.9
1.0% Pd_1/CALB-P	73.2	8.6	5.3	13.1
3.5% Pd_1/CALB-P	58.5	14.1	9.4	17.6
ADH	59.1	16.4	10.1	14.2
1.5% Pd_1/ADH-P	47.2	21.6	19.0	12.7
CAT	75.6	0.5	8.3	15.6
1.9% Pd_1/CAT-P	66.5	6.3	14.7	12.4
GOx	65.5	18.1	9.0	7.8
1.0% Pd_1/GOx-P	59.8	23.9	16.7	17.2
Lac	64.1	15.3	6.1	14.3
0.5% Pd_1/Lac-P	59.1	17.9	9.0	12.9

Note: The analysis was processed by software CD Pro, corresponding to the CD spectra in **Supplementary Figure 20**.

Supplementary Table 7 Cross-coupling reactions of alkyl electrophile and alkylboron reagents catalyzed by different metal-based catalysts.

Ent-ry	Alkyl electrophile	Alkylboron reagent	Catalyst	Condition	Conv. (%)	Rate (mmol h^{-1} g_{metal}^{-1})	Ref.
1	$CH_3(CH_2)_5I$	$(9\text{-}BBN)(CH_2)_7CH_3$	$Pd(PPh_3)_4$	60 ºC, dioxane, 24h	64	8	[10]
2	$CH_3(CH_2)_3I$	$(9\text{-}BBN)(CH_2)_7CH_3$	$[(^{Me}N_2N)Ni\text{-}Cl]$	80 ºC, dioxane, 15h	50	11	[11]
3	$CH_3(CH_2)_{11}Br$	$(9\text{-}BBN)(CH_2)_7CH_3$	$Pd(OAc)_2$, PCy_3	r.t., THF, 16h	85	12	[12]
4	$NC(CH_2)_6Br$	$(9\text{-}BBN)(CH_2)_{10}CO_2Me$	$Pd(OAc)_2$, PCy_3	r.t., THF, 16h	81	12	[12]
5	$Ph(CH_2)_4Br$	$(9\text{-}BBN)(CH_2)_7CH_3$	$[(MeN_2N)Ni\text{-}Cl]$	80 ºC, dioxane, 24h	68	10	[11]
6	$Ph(CH_2)_3Br$	$(9\text{-}BBN)(CH_2)_6CH_3$	Pd-PEPPSI-IPr	r.t., dioxane, 16h	87	13	[13]
7	$CH_3(CH_2)_{11}Br$	$(9\text{-}BBN)(CH_2)_6CH_3$	$Pd(OAc)_2$, phosphaadam-antane ligand	r.t., THF, 24h	93	9	[14]
8	$CH_3(CH_2)_{11}Cl$	$(9\text{-}BBN)(CH_2)_7CH_3$	$Pd_2(dba)_3$, PCy_3	90 ºC, dioxane, 48h	77	3	[15]
**9**	**$CH_3(CH_2)_5Br$**	**$(9\text{-}BBN)(CH_2)_7CH_3$**	**Pd_2/CALB-P**	**r.t., H_2O:THF (4:1), 2h**	**86**	**284**	**This work**

Supplementary Table 8 Binding free energy G_{bind} of CALB&1-bromohexane and CALB&1-bromododecane complexes using MM/GBSA method. The van der Waals contribution, electrostatic contribution, polar solvation term (EGB), and non-polar solvation term (ESurf) were also listed.

Energy (kcal/mol)	CALB&1-bromohexane	CALB&1-bromododecane
Van der Waals	-22.6 ± 1.4	-35.9 ± 2.3
Electrostatic	-0.89 ± 1.0	-0.9 ± 0.6
E_{GB}	5.6 ± 1.1	6.6 ± 0.8
E_{Surf}	-3.3 ± 0.1	-5.0 ± 0.2
ΔG_{bind} MM-GBSA	-21.2 ± 1.6	-35.1 ± 2.4

Supplementary References

[1] Sayle, R., Milnerwhite, E. Rasmol-Biomolecular Graphics for All. *Trends Biochem. Sci.* **20**, 374-376 (1995).

[2] Hess, B., Kutzer, C., van der Spoel, D., Lindahl, E. GROMACS 4: Algorithms for highly efficient, load-balanced, and scalable molecular simulation. *J. Chem. Theory Comput.* **4**, 435-447 (2008).

[3] Jorgensen, W. L., Chandrasekhar, J., Madura, J. D. Comparison of simple potential functions for simulating liquid water. *J. Chem. Phys.* **79**, 926-935 (1983).

[4] Uppenberg, J., Hansen, M. T., Patkar, S., Jones, T. A. The sequence, crystal structure determination and refinement of two crystal forms of lipase B from Candida antarctica. *Structure* **2**, 293-308 (1994).

[5] Morris, G. M. et al. AutoDock4 and AutoDockTools4: Automated docking with selective receptor flexibility. *J. Comput. Chem.* ***30***, 2785-2791 (2009).

[6] Miller, III B. R. et al. MMPBSA. py: an efficient program for end-state free energy calculations. *J. Chem. Theory Comput.* **8**, 3314-3321 (2012).

[7] Bussi, G., Donadio, D., Parrinello, M. Canonical sampling through velocity rescaling. *J. Chem. Phys.* **126**, 014101 (2007).

[8] Parrinello, M., Rahman, A. Crystal structure and pair potentials: A molecular-dynamics study. *Phys. Rev. Lett.* **45**,1196 (1980).

[9] Humphrey, W., Dalke, A., Schulten, K. VMD: visual molecular dynamics. *J. Mol. Graphics* **14**, 33-38 (1996).

[10] Ishiyama, T., Abe, S., Miyaura, N., Suzuki, A. Palladium-catalyzed alkyl-alkyl cross-coupling reaction of 9-alkyl-9-BBN derivatives with iodoalkanes possessing β-hydrogens. *Chem. Lett.* **21**, 691-694 (1992).

[11] Di Franco, T., Boutin, N., Hu, X. Suzuki-Miyaura cross-coupling reactions of unactivated alkyl halides catalyzed by a nickel pincer complex. *Synthesis-Stuttgart* **45**, 2949-2958 (2013).

[12] Brenstrum, T. et al. Phosphaadamantanes as ligands for palladium catalyzed cross-coupling chemistry: Library synthesis, characterization, and screening in the suzuki coupling of alkyl halides and tosylates containing β-hydrogens with boronic acids and alkylboranes. *J. Org. Chem.* **69**, 7635-7639 (2004).

[13] Valente, C. et al. High yielding alkylations of unactivated sp^3 and sp^2 centres with alkyl-9-

BBN reagents using an NHC-based catalyst: Pd-PEPPSI-IPr. *Chem. Commun.* **6**, 735-737 (2008).
[14] Chen, X., Engle, K. M., Wang, D. H., Yu, J. Q. Palladium(II)-catalyzed C-H activation/C-C cross-coupling reactions: versatility and practicality. *Angew. Chem., Int. Ed.* **48**, 5094-5115 (2009).
[15] R. Chinchilla, C. Nájera, Recent advances in Sonogashira reactions. *Chem. Soc. Rev.* **40**, 5084-5121(2011).
